# Behavioural drivers of child feeding during and after illness in the Democratic Republic of the Congo: results from a qualitative study through the lens of behavioural science

**DOI:** 10.1017/S136898002300294X

**Published:** 2023-12-27

**Authors:** Emily Zimmerman, Madeline Kau, Riantsoa Kanto Najaina Tovohasimbavaka, Augustin Ngandu, Didier Mbayi Kangudie, Lynn Van Lith, Radha Rajan, Danielle Naugle, Lisa Sherburne

**Affiliations:** 1 ideas42, 80 Broad St, Floor 30, New York, NY 10004, USA; 2 Johns Hopkins Center for Communication Programs, 111 Market Place, Suite 310, Baltimore, MD 21202, USA; 3 JSI Research and Training Institute, 44 Farnsworth Street, Boston, MA 02210, USA

**Keywords:** Behavioural science, Complementary feeding, Child illness, Democratic Republic of the Congo

## Abstract

**Objective:**

For young children experiencing an illness, adequate nutrition is critical for recovery and to prevent malnutrition, yet many children do not receive the recommended quantities of food during illness and recuperation. Our research applied a behavioural science lens to identify drivers of feeding behaviours, including barriers inhibiting caregivers from following the feeding guidelines.

**Design:**

In 2021, we conducted qualitative research informed by the behavioural design process. Data from in-depth interviews and observations were analysed for themes.

**Setting:**

Research was conducted in South Kivu, Democratic Republic of the Congo.

**Participants:**

Research participants included caregivers of young children, other family members, health workers and other community members.

**Results:**

Five key findings about behavioural drivers emerged: (1) poverty and scarcity impose practical constraints and a cognitive and emotional burden on caregivers; (2) health providers are distracted and discouraged from counselling on feeding during sick visits; (3) a focus on quality and hesitations about quantity obscure benefits of feeding greater amounts of available foods; (4) perceptions of inappropriate foods limit caregivers’ choices; and (5) deference to a child’s limited appetite leads to missed opportunities to encourage them to eat.

**Conclusions:**

Each of these behavioural drivers is triggered by one or more addressable features in caregivers’ and health workers’ environment, suggesting concrete opportunities for programmes to support caregivers and health workers to improve feeding of young children during illness and recovery. In other settings where these features of the environment are similar, the insights and programming implications are likely to translate.

For young children experiencing an illness, adequate nutrition is critical for recovery and to prevent malnutrition^(1)^. Children’s growth deteriorates rapidly during and after illness if foods and feeding practices do not meet the additional nutrient requirements associated with illness. The global guidance is for children aged 6–23 months to continue to eat and breastfeed as much as possible during illness and to consume more than usual in the 2 weeks following illness^(2)^. Despite the importance of these behaviours, relatively less research and programming has focused on them, compared with other complementary feeding behaviours^(3)^.

Although published data on feeding during and after illness are limited, the available data suggest that many children are not fed according to the guidelines, particularly in low-resource settings. A recent literature review analysing trends from available population-based surveys across fifteen African countries found that approximately half or more of children under 5 years of age received less or no food during instances of diarrhoea. The review also noted that no countries are on track with increasing trends in feeding during diarrhoea^(3)^.

In the South Kivu province of the Democratic Republic of the Congo (DRC), 42 % of children under 5 years of age receive a lot less or no liquids and 22 % receive a lot less or no food during instances of diarrhoea, as compared with times of good health^(4)^. In a qualitative study in rural South Kivu, half of the mothers interviewed described breastfeeding their young child less than usual during illness^(5)^. In the same study, only 40 % of mothers reported their child consumed more breast milk than usual during recovery and only 4–6 % reported increasing the amount of complementary foods offered during recovery^(5)^.

These gaps in complementary feeding exist within a broader context of poverty, food insecurity and malnutrition in the DRC. Over 25 million Congolese people experienced acute food insecurity in 2023^(6)^. As of 2018, 73 % of the population was estimated to live in extreme poverty^(7)^. In the same year, 42 % of children under 5 years were chronically malnourished and 6·5 % of children under 5 years were acutely malnourished^(7)^. In the South Kivu province, young children’s diets are frequently inadequate. A 2021 study found that 52 % of young children in rural communities received the minimum recommended meal frequency, 21 % received minimum dietary diversity and 26 % received a minimum acceptable diet in the previous 24 hours^(8)^.

The country has also made a number of global and regional commitments to improve nutrition outcomes^(9)^. The National Nutrition Program (PRONANUT) within the Ministry of Health leads the development and implementation of nutrition policy, in coordination with a range of multisectoral government and non-governmental partners supporting health, agriculture, gender, and food security activities^(10)^. In the context of these ambitious aims and robust ongoing programming, there is potential for innovative social and behaviour change (SBC) programme and service approaches to improve complementary feeding behaviours and reduce malnutrition. SBC seeks to improve and sustain changes in nutrition behaviours by understanding and addressing individual, social, and structural factors that influence dietary and caring practices. In the DRC, feeding during and after illness deserves urgent attention as a priority for SBC^(11)^ because of the frequency of childhood illness and the large gap between current practice and the recommendations. Addressing this gap would contribute significantly to nutrition outcomes. Increasing feeding during and after illness also holds promise as an area of focus for SBC because following the global nutrition guidance for these behaviours does not require children to be fed specific foods. Any nutritious family foods, if they are appropriately prepared for the child’s age, can be beneficial. As a result, these behaviours are more feasible (relative to other complementary feeding behaviours) for household members to adopt even within significant resource constraints.

This article describes research conducted in South Kivu, DRC, to illuminate the drivers of caregivers’ feeding choices and behaviours during and after illness, for the purpose of informing and strengthening health and nutrition programmes and services^(11)^ to improve child feeding during and after illness. The research was conducted as part of a behavioural design process to develop SBC solutions – described elsewhere^(12)^ – to improve nutrition outcomes for infants and young children in the DRC. This article provides a more in-depth exposition of the research methods and results described in a programmatic research brief^(13)^.

## Methods

### Study aim and design

This study’s aim was to identify behavioural drivers that may influence feeding of young children in the DRC during and after illness and to identify features of the context shaping caregivers’ and health workers’ behaviour that services and programmes can address to improve feeding. We conducted a cross-sectional qualitative research study comprised of in-depth interviews with caregivers and other stakeholders who may influence or have a perspective on child feeding behaviours and observations of clinical consultations where child feeding may have been discussed. This research served as the diagnosis stage of a behavioural design process, which employs a systematic approach to apply insights from behavioural science to solution development^(14)^. The research was followed by collaborative design activities to develop solutions that modify features of caregivers’ and health providers’ context to generate SBC.

### Study setting

Field research was conducted in April and May 2021 in health facilities providing primary care services and in communities in 4 Aires de Santé (health areas) within the health zones of Katana and Mubumbano, South Kivu. Health areas and health facilities were selected by local programme staff in collaboration with Provincial Health Division officials to represent a range of rural and peri-urban geographies (two rural and two peri-urban), facility types (one health centre and three health posts) and health facility performance levels (one low-performance, two of average performance and one higher performance) as identified by the Médecin Chef de Zone, the local Ministry of Health official for each health zone. The selected health areas also had ongoing programmes addressing child nutrition through which SBC solutions might be implemented.

### Study participants

We conducted qualitative research to identify drivers of the behaviours of focus: caregivers continue to feed young children as much as possible during illness and feed more than usual in the 2 weeks following illness. This included fifty-eight in-depth interviews with individuals with diverse perspectives on these behaviours. Participants included mothers and fathers of children aged 6–23 months who live in the same household with the child, paternal grandmothers of children aged 6–23 months living in the same health area, community leaders including village chiefs, traditional healers, and women’s group leaders, and facility-based health providers and community health workers (CHW) who attend to sick children and had held their role in the study community for at least 1 month. We also conducted observations of sick and well-child consultations in health facilities in which nutrition, including child feeding recommendations, might have been discussed, including postnatal, immunisation and sick child visits. Table 1 summarises participants and activities.

**Table 1:** Research participants and activities

Participants	Number	Activities
Mothers	24	In-depth interviews
Fathers	7	In-depth interviews
Grandmothers	8	In-depth interviews
Facility-based health providers	7	In-depth interviews
Community health worker	4	In-depth interviews
Community leaders	8	In-depth interviews
Health facility consultations	10	Observations

### Processes

Client participants and facility-based health providers were recruited for participation in observations and health provider interviews through convenience sampling at health facilities during off-peak hours, in coordination with the health facility supervisor at each selected facility. CHW were identified by facility-based providers and local governmental authorities and invited to participate at a time and location of their choosing. Community leaders were identified by local project staff and invited to be contacted by the study team at a time and location of their choosing. Caregivers were identified by CHW and local project staff and invited to be visited by a member of the study team at their home or in another location of their choosing. For all participants, local staff and partners used a short recruitment script to obtain permission to put potential participants in touch with interviewers, and the interviewers followed a standard recruitment script and informed consent process. Interviews were conducted outdoors as a precaution against COVID-19 transmission, in a private space outside earshot of other people. Each participant was offered a small non-monetary token of appreciation for their participation.

Interviews and observations investigated hypothesised drivers of feeding generated through a review of literature on feeding sick and recovering children and behavioural science principles of anticipated relevance (e.g. schemas^(15)^, attention^(16)^ and the impacts of scarcity^(17)^). Data collection instruments were also informed by the Extended Parallel Process Model, which posits that behaviour is influenced by both perceived threat and perceived efficacy, and an individual is most likely to act when both are high^(18)^.

Interviews followed semi-structured interview guides and were conducted by two trained interviewers who live in South Kivu. Each interview was conducted in Kiswahili, Mashi, or French (according to the participant’s preference) and recorded, transcribed, and translated to English for analysis. Translated transcripts were back-checked by the interviewers to ensure participants’ meaning was accurately captured. Observations followed a structured note-taking guide. Copies of the research instruments, in French, are available in the Appendices.

The study plans and research instruments were reviewed and approved by the Johns Hopkins Bloomberg School of Public Health Institutional Review Board (JHSPH IRB) (IRB no. 14879) and by the University of Kinshasa School of Public Health Ethics Committee (approval no. ESP/CE/08/2021). Study plans and instruments were also discussed with the South Kivu-based research firm Research Initiatives for Social Development to ensure clarity and conciseness and to underscore practices necessary to maintain ethical conduct during the research process. Approval to conduct the research was received from the DRC Ministry of Health.

### Analysis

We employed thematic analysis using a combination of inductive and deductive analytical techniques, drawing from the approach outlined by Braun and Clarke^(19)^. The research team generated an initial codebook of thirty thematic codes from the hypotheses developed prior to data collection. Codes were adjusted throughout the coding process. Four members of the research team participated in coding, and multiple coders coded four transcripts from a range of respondent segments and discussed and resolved discrepancies.

Authors then assessed the coded evidence against the hypotheses, evaluating the strength of the evidence supporting or refuting each hypothesis and documenting emergent themes that were not captured in the initial hypotheses. Multiple reviewers independently identified a subset of findings about drivers of feeding practices during and after illness that were most strongly supported by the evidence. Authors participating directly in analysis include a Congolese national with knowledge of infant and young child feeding. Authors discussed and refined these findings in concert with local interviewers and project and government stakeholders to ensure proper understanding of the results in context.

## Results

Five key findings about feeding during and after illness emerged from the analysis.

### Poverty and scarcity impose practical constraints and an emotional and cognitive burden

Poverty and food insecurity constrain what families can offer their children. Caregivers, community leaders, CHW and providers all described severe constraints on the quantity and type of food that affect nearly all families in their communities. Caregivers noted that all family members, including young children, frequently eat smaller quantities, fewer meals per day and less nutritious food than they would prefer. Caregivers could identify nutritious foods that they had in the home but mentioned that they could not afford to purchase the preferred types of ‘good’ food and as much food as they wanted for their children, both during and outside of illness. When asked about how they care for their young children during illness and recuperation, caregivers mentioned their financial limitations and constraints quickly and without prompting. Nearly, all caregivers repeatedly expressed feelings of frustration and discouragement with these constraints.

*It’s good to give extra food to convalescents if you can afford it. But I don’t have much money. You can’t give a child food you don’t have just because he’s convalescing. We are not going to steal for him.*—Mother of a 6–11-month-old child, rural community

*We eat together the small quantity available…we take some water and we sleep.*—Father of a 6–11-month-old child, rural community

*The major challenge we face is the lack of money. The population is poor and everything we give as recommendations requires financial means.* —CHW, rural community

### Health providers do not counsel on child feeding during sick child visits due to distraction and a sense of discouragement

Counselling on feeding is a component of the Integrated Management of Childhood Illness (IMCI) protocol^(20)^ and the counselling protocol for health providers during sick child visits in the DRC. However, interviews suggested that health providers do not consistently discuss feeding during sick child consultations. When reflecting on what they do and discuss in these visits, providers first described medical treatment. They noted that they sometimes discuss food hygiene or nutritional needs for the child’s particular illness but did not mention counselling on the optimal quantity of food. Observations of consultations were consistent with providers’ reflections: of six sick child visits observed, only three touched on nutrition at all; in these consultations, providers promoted dietary diversity generally and mentioned specific recommended foods but did not counsel on quantity. When asked about the feeding guidelines, providers noted that young children should continue to eat during illness.

Interviews and observations suggested that providers typically do not counsel on feeding after illness at all. When asked in interviews to describe what they discuss in sick visits, no providers mentioned counselling on feeding for the recovery period unprompted. When prompted, some providers noted that recovering children should be fed similar foods in similar amounts to before illness, suggesting that they may misunderstand the optimal feeding practice of increasing food in the 2 weeks following illness. Feeding after illness was not discussed in any of the sick child consultations observed.

*[They should feed] in the same way as before the disease so that the child does not relapse.*—Provider, peri-urban health facility

Some providers expressed hesitation to counsel on feeding and frustration at being expected to give advice that they do not believe caregivers can put into practice due to their limited resources. They described instances of caregivers responding negatively to nutrition counselling and protesting that it is unfair for providers to advise them to feed their children in a certain way when they do not have the food to do so.

*[When we advise on feeding,] they react by asking the question: if we can’t afford it, what are we going to do?*—Provider, peri-urban health facility

*We must be content with giving advice [on feeding] while being aware that they will not apply it because they do not have the means.*—Provider, peri-urban health facility

### A focus on perceived quality of foods and hesitations about quantity obscure benefits of feeding more of available foods

Caregivers and other community members described a need for the child to regain strength lost during illness by eating specific foods, such as meat and dairy products, that they associate with strength. Families in South Kivu can rarely afford or access these specific foods, and caregivers sometimes considered equally (or even more) nutritious but more affordable and consistently available local options such as small fish not to be as good. Caregivers noted that they did not see much value to the child’s recovery in feeding more of available foods.

*Meat, eggs, milk, fish … these are foods that can help him to recover quickly.*—Community leader, rural community

*Fretins [small dried fish] are not nutritious at all.*—Father of a 12–23-month-old child, rural community

*You can’t add what you don’t have… there are no more cows; the milk is on the market but there are no means. It all depends on the means.*—Grandmother of a 6–11-month-old child, peri-urban community

Caregivers also mentioned concerns about feeding too much. They also noted that increasing the quantity of food too rapidly as the child recovers can be harmful. Some described extra food as unhealthy or as detrimental to recovery. Others expressed concern that more food would lead to gluttony or make the child accustomed to receiving more than they can provide. Most caregivers expressed that the amount of food a child consumes after illness should be increased only slowly and gradually. Some caregivers described this as necessary to avoid outpacing the child’s appetite. Others described it as necessary to avoid a relapse of the illness, even if appetite was strong.

*It is necessary to give him the right food. Not a lot of food.*—Grandmother of a 6–11-month-old child, rural community

*It’s not a good idea to bombard your baby with food because his body is not yet in good shape. This can cause digestive problems or lead to other diseases.*—Mother of a 6–11-month-old child, rural community

*I told her not to give the child the porridge twice a day because if we get him used to it at this frequency, tomorrow he will miss it.*—Father of a 6–11-month-old child, rural community

*Children should not be taught to gluttony; they should be given an average amount that they can finish and avoid waste. Gluttony is a sin.* —Mother of a 12–23-month-old child, peri-urban community

Caregivers commonly expressed concern that large meals for sick and recovering children might lead to wasted food. However, upon probing, they noted that food was rarely wasted even if the child did not eat what was offered; this food was either given to other children or saved for later.

### Perceptions of inappropriate or low-quality foods limit choices

Caregivers commonly characterised their situation as constrained, expressing that they felt powerless to offer what they knew their child needed, with keen attention to the foods they felt unable to offer. Some foods were described as unavailable or unaffordable and some as likely to be refused by the child. Other foods were described as harmful, either for all young children or specifically for those who are sick or recovering. While caregivers and other community members did not consistently describe the same foods as harmful, nearly all of the foods most commonly available in South Kivu were described by some respondents as bad for young children.

*Beans and vegetables are bad after the disease except for amaranths…When the child has diarrhea, you cannot give him sombé [cassava leaves] or bean leaves.*—Mother of a 6–11-month-old child, rural community

*The bad foods in my opinion are the vegetables prepared without oil…vegetables prepared only with water are not good for children.*—Mother of a 12–23-month-old child, peri-urban community

*If you give beans or bananas to a sick baby it is a waste of time…he can’t eat them.*—Father of a 6–11-month-old child, peri-urban community

*Everything depends on the disease…for cough, cereals are not good.*—Women’s group leader, peri-urban community

Interviews with providers did not suggest that they commonly held mistaken perceptions that certain foods are inappropriate; when asked what sick and recovering children could eat, they cited only a few ‘bad’ foods. Observations of sick child consultations also suggested that providers do not directly advise against many foods. However, caregivers commonly reported that they had learned about harmful foods from providers, suggesting that by emphasising specific (often unavailable) foods as beneficial, providers may inadvertently signal to caregivers that *only* those foods are acceptable and high quality.

Interviews with caregivers also suggested that they may overestimate the cost and effort required to feed children well during and after illness. They commonly noted that offering extra foods would be too costly but were rarely able to estimate specific costs, especially the cost of small amounts of the most affordable staple foods, including those their families already purchase or grow and prepare. Some caregivers also estimated that special feeding after illness should last far longer than the recommended 2 weeks.

*The child needs special care to avoid relapse, to recover his health. We must buy him energy foods such as corn, sorghum and soybean flour that we can mix to make his porridge; we can also get him fish, milk, meat and others. This care can go up to 4 months.*—Mother of a 12–23-month-old child, peri-urban community

### Caregivers miss opportunities to encourage the child to eat more when appetite is limited

Caregivers noted, often with frustration, that what the child can or will consume during and after illness is out of their control. They described changes in a child’s appetite as the primary signals that tell them when a child has fallen ill and when they are beginning to recover. Only a small minority of caregivers mentioned specific strategies to encourage children to eat when their appetite is low during illness; among those caregivers, ‘forcing’ or offering the child’s favourite foods were most cited.

*In case of illness, he doesn’t eat. Even if I force him to swallow something, it is useless.*—Mother of a 6–11-month-old child, rural community

Caregivers did not mention small, frequent portions of food as a strategy to encourage a child to eat more when appetite is limited. When prompted, some actively objected to this tactic. Caregivers also noted in interviews that grandmothers, aunts and older siblings frequently care for children when the mother is occupied with other tasks, both during and outside times of illness. Because mothers in South Kivu generally have most or all of a family’s cooking responsibilities, cooked foods are generally unavailable when the mother is not at home.

*[Giving small, frequent portions would be a problem] because it can stunt the growth of the child and swell the belly.*—Grandmother of a 6–11-month-old child, rural community

*[After illness,] the frequency should not change or he will be vomiting if you give him food all the time.*—Mother of a 12–23-month-old child, peri-urban community

Mothers described knowing that they should continue to breastfeed during and after illness but did not mention efforts to increase consumption of breast milk during these times. Mothers noted that they nurse when the child asks or when their breasts feel full, but most did not describe offering breast milk more frequently or other tactics to increase consumption during or after illness.

*From birth to 7 months of age I decide how to breastfeed the child… After this age when the child needs it, he approaches and asks for the breast milk… I don’t decide anything for his breastfeeding, but when he wants it he asks for it and I give it to him.*—Mother of a 12–23-month-old child, peri-urban community

## Discussion

The research identified five key drivers of feeding choices and behaviours for young children during and after illness in South Kivu. Poverty and scarcity impose immense practical constraints on what caregivers can offer their young children, as well as a cognitive and emotional burden that at times may obscure the options that remain within their control. Health providers sometimes fail to counsel appropriately on feeding during sick visits when they are focused on medical treatment and when they doubt that caregivers can put their guidance into practice due to their limited means. Caregivers and other community members perceive certain foods (which are often less available and affordable to them) to be of greatest value during and after illness and do not perceive much value to feeding more of other foods, including affordable staple foods. They also express concerns that over-feeding might undermine the child’s recovery or negatively influence the child’s later expectations or behaviour. Perceptions that certain foods are low quality or inappropriate further limit the choices families perceive to be available to them. Finally, caregivers sometimes miss opportunities to encourage children to eat more when their appetite is limited.

This research builds on prior nutrition, complementary feeding and behavioural science research. It also suggests opportunities for programmes and services to support caregivers and health workers to improve child feeding during illness and recovery by addressing the underlying behavioral and contextual drivers of their choices. These insights, and their implications for programme design, may translate to other settings where the underlying drivers are similar.

The constraints imposed by food insecurity have been extensively documented^(21)^. Prior behavioural science research has found that in addition to the severe practical challenges they impose for families, circumstances of chronic scarcity impose a cognitive burden^(17)^, which is exacerbated by drawing attention to the condition of scarcity^(22)^. Our research builds upon this research by demonstrating how poverty and food insecurity weigh particularly heavily on caregivers in South Kivu when they consider how they can care for their sick and recovering children. This finding suggests that programmes to reduce food insecurity and boost families’ financial stability may have value not only in preparing them to meet their practical needs but also by easing the cognitive and emotional burden that can negatively impact decision-making. Programmes and services can also respond to these conditions of scarcity by working with caregivers to expand their options for increasing feeding within the constraints they face, for example, by identifying and elevating the locally available, affordable, and nutritious foods that the family already eats and that can be fed to a young child. The findings suggest that programmes can also reduce the cognitive and emotional impacts of scarcity by redirecting caregivers’ attention away from what they cannot do and towards what is within their control.

Prior research has described the influence of norms and customs on caregivers’ perceptions of how and which foods young children should be fed, including perceptions of appropriate and inappropriate or unhealthy foods^(23–26)^. This study builds upon the prior research by describing how these perceptions further limit caregivers’ perceived options for their sick and recovering children. It also describes how nutrition counselling by health workers that emphasises specific recommended foods for sick and recovering children may inadvertently reinforce perceptions that other foods are inappropriate or not worthwhile during these times. Prior studies have also documented fears that certain feeding practices might predispose children to have certain expectations and influence their later behaviour, such as a fear that if a child becomes accustomed to eating eggs and other animal-source foods, they might steal or beg for those foods^(27–29)^. Fears about the impacts of eggs or other animal-source foods persist despite an absence of evidence that feeding children those foods has any impact on children’s later behaviour^(27)^. This study identified analogous concerns related to the perceived effects of a different feeding practice: increasing quantity or frequency of feeding during and after illness. Caregivers expressed concern that it might lead to unrealistic expectations from children about how much or how often they would receive food during times of good health. Programmes and services can address such misconceptions about inappropriate foods and feeding practices directly. They can also emphasise key messages about the importance of quantity during illness and recovery.

The Extended Parallel Process Model posits that individuals are most likely to act when they perceive both threat and efficacy to be high^(18)^. Our research illuminates several reasons underlying caregivers’ low self-efficacy (i.e. perceived ability) to feed their children well during and after illness, including perceptions about the quality of the foods they can access and the extent to which they can encourage a child to eat more when appetite is limited. It also reveals doubts about the response efficacy (i.e. perceived effectiveness) of feeding more, rooted in perceptions that over-feeding may lead to negative effects on recovery and unwanted effects on future feeding. Programmes and services can encourage caregivers to act by addressing these underlying drivers of low self-efficacy and response efficacy. Further research could quantify caregivers’ levels of efficacy regarding feeding children during illness and recovery, test the associations between these factors and the behaviour of increased child feeding during and after illness and measure the impact of programmatic interventions aimed at improving self-efficacy and response efficacy.

Prior research has found that health workers do not consistently counsel on nutrition and feeding during sick child consultations^(1,3)^. Our research builds upon this by explaining how doubts about the caregiver’s ability to afford food perceived to be good for the child discourage providers from bringing up feeding guidance. Several behavioural tendencies may help to explain providers’ avoidance of the topic, including a tendency to avoid negative information and feedback even when it could be useful^(30)^ and a tendency to set low expectations to reduce anxiety about one’s ability to follow through^(31)^. Services and programmes can address this by orienting providers towards nutrition counselling that caregivers can regularly put into practice, rather than towards specific foods which may be inaccessible or unaffordable.

Prior research in the DRC has found that caregivers often do not encourage their young children to eat during times of illness^(32)^. Research has also described caregivers’ perceptions of appetite and identified characteristics of caregivers that may lead them to be less informed about their children’s appetite cues^(33)^. Our research builds on this by describing several underlying reasons why caregivers fail to encourage their children to eat when appetite is limited, including a perception that the child’s limited appetite is fixed, a limited repertoire of concrete and accessible strategies for encouraging a child to eat, and concerns about over-feeding and food waste. Programmes and services can address each of these distinct challenges by boosting financial stability and food security, by reframing what is possible and desirable, by teaching new skills and by directing attention towards small increases in feeding of locally accessible and affordable foods.

This research investigated an under-explored area of child nutrition^(3)^ and behaviours likely, if improved, to generate meaningful impact on child nutrition outcomes. A diverse range of in-country and global stakeholders supported research activities, bringing technical and contextual expertise in child nutrition, SBC programming, social and behavioural science, and local programming needs. It captured the perspectives of different types of caregivers (mothers, fathers and grandmothers) as well as facility- and community-based health workers; these perspectives were triangulated against direct observation of clinical consultations. By bringing together these different perspectives, we identified specific behavioural and contextual drivers of caregivers’ behaviour. Although this qualitative study cannot definitively assert the behavioural and psychological mechanisms described above, prior behavioural science research helped us to interpret the evidence collected and anticipate how solutions based on this evidence are likely to be received.

This research was conducted as part of a behavioural design process, through which solutions were developed to address each of the key findings described above. These solutions, described in a programmatic brief^(12)^, and intended for implementation through programmes focused on increasing food security, aim to support families to set achievable goals for feeding during and after illness, consider additional affordable and nutritious local foods, build skills and confidence to overcome limited appetite and celebrate each bite the sick and recovering child takes as a small victory. The solutions include counselling aids and reminders for healthcare providers, a facilitated peer exchange on tactics to encourage young children to eat when appetite is limited and card-based activities facilitated by a CHW during visits to families of sick children.

## Conclusion

This study sought to better understand the behavioural drivers influencing feeding of young children during and after illness. Through in-depth individual interviews and observations, it describes how factors in health facilities, households and communities influence how young children are fed during these times. It was conducted as part of a behavioural design process to develop SBC solutions to improve nutrition outcomes for infants and young children in the DRC. Similar drivers may be likely to arise in settings where families cannot consistently access or afford foods they believe to be nutritious for their young children, where feeding may not be adequately discussed during sick child consultations, where nutrition counselling and materials emphasise specific foods that may not be accessible to families, where some affordable, nutritious, locally available foods are undervalued or considered unhealthy, inappropriate, or less beneficial for young children, and where children are not commonly encouraged to eat when their appetite is limited. By addressing these behavioural and contextual drivers of health workers’ and caregivers’ choices, services and programmes have potential to generate meaningful impact on child health and nutrition outcomes, even in highly resource constrained settings.

## Supporting information

Zimmerman et al. supplementary material Zimmerman et al. supplementary material

## Supplementary material

For supplementary material accompanying this paper visit https://doi.org/10.1017/S136898002300294X
