# Supplementary material for: Behavioural drivers of child feeding during and after illness in the Democratic Republic of the Congo: results from a qualitative study through the lens of behavioural science
Source: Public Health Nutr. 2023 Dec 27;27(1):e80. doi: 10.1017/S136898002300294X (PMC10966839; doi:10.1017/S136898002300294X)
Supplement: Zimmerman et al. supplementary material [file S136898002300294Xsup001.docx]

**Interview Guide : Community Leaders**

**Rôle**

- Pouvez-vous me parler de votre rôle dans cette communauté ?

**Alimentation générale**

- Pourriez-vous décrire ce que mange une famille typique de votre communauté au cours d'une journée typique ?
  - Comment cela pourrait-il changer en fonction du jour, de la semaine, du mois, de la saison ?
    - Quand peut-on se procurer davantage de nourriture ?
    - Quand y a-t-il moins de nourriture disponible ?
  - Où les familles trouvent-elles généralement leur nourriture *(c'est-à-dire au marché, dans les stands le long des routes, sur leurs propres terres, chez les voisins/ONG, dans la nature)* ?
    - Qui fait les courses ? / Qui collecte la nourriture ?
    - Quand font-ils leurs achats ou leurs collectes ?
  - Quand les familles mangent-elles généralement ?
    - Combien de fois par jour les membres de la famille mangent-ils ?
- Comment nourrir un enfant de 6 à 12 mois ?
  - Y a-t-il quelque chose de difficile ? Intéressant ?
- Pouvez-vous décrire comment les enfants de 6 à 12 mois mangent habituellement ?
  - Que mangent-ils ?
  - Quand mangent-ils ?
  - Quel est l'aliment qui nourrit le plus un bébé ?
    - *Sondez : Lait maternel, bouillie, fufu, un autre aliment*
- Comment savoir si un bébé (à cet âge) mange bien ?
- Qui, dans une famille, décide généralement de ce que le bébé doit manger à cet âge ?
  - La mère reçoit-elle des conseils de quelqu'un ?
  - Quel est le rôle du mari ? Belle-mère ? Quelqu'un d'autre ?
- Avez-vous donné aux familles des conseils ou des instructions sur la façon de nourrir un bébé ?
  - *Si oui :*
    - À qui avez-vous donné des conseils ?
    - Qu'est-ce qui vous a incité à leur donner des conseils ?
    - Qu'avez-vous dit ?
  - *Si non :* pourquoi *n*'avez-vous jamais donné de conseils à ce sujet ?

**Maladie**

- Dans cette communauté, quelle est la fréquence des maladies ?
  - Quel type de maladie?
  - Quelle saison?
  - Qui tombe généralement malade?
  - Que font les collectivités pour lutter contre ces maladies, le cas échéant ?
  - Si quelqu'un tombe malade, les personnes extérieures au foyer en entend-on généralement parler ?
  - Les gens mangent-ils généralement différemment pendant ou après la maladie ?
    - *Si oui :* comment se nourrissent-ils différemment ?
- Est-il fréquent que les enfants de cette communauté tombent malades entre 6 mois et 1 an ?
  - Quelles sont les maladies les plus courantes ?
  - Quelles sont les causes de ces maladies ?
  - Certaines maladies sont-elles plus fréquentes à certaines périodes de l'année ? Lesquelles?
  - Que font les familles lorsque leur enfant est atteint de ces maladies ?
  - Si un enfant tombe malade, les personnes extérieures au foyer en entend-on parler ? Pourquoi ou pourquoi pas?
- Lorsqu'un enfant a *[demandez pour chacun : diarrhée, toux, fièvre/corps chaud]*, que fait généralement la famille ?
  - *Sonder : observer attentivement, rester à la maison, changer d'alimentation, demander conseil à des parents ou amis, aller dans un établissement de santé, guérisseur traditionnel, médecine, autres actions pour le confort physique*
  - Qui, dans la famille, décide généralement de ce qu'il faut faire lorsqu'un enfant de cet âge tombe malade ?
- Vous êtes-vous déjà fait dire ou avez-vous remarqué par vous-même qu'un bébé était malade ?
  - Comment avez-vous su qu'ils étaient malades ?
  - Qu'avez-vous fait?
- Avez-vous déjà parlé à un membre de la communauté de ce qu'il faut faire lorsque son bébé est malade ?
  - Quand?
  - Qu'avez-vous dit?
  - Qu'est-ce qui vous a incité à donner ce conseil ?
- Que disent les gens de votre communauté au sujet du centre de santé local ? A propos des prestataires?
  - Que pensez-vous du centre de santé local ? A propos des prestataires?

**Recouvrement**

- Lorsqu'un enfant de 6 à 12 mois est malade, combien de temps dure-t-il généralement ?
  - Comment savoir quand elle s'arrête ?
    - *Sondez : Si l'enfant pleure plus/moins, changement d'appétit de l'enfant, selon le fournisseur de soins, selon la mère*
    - *Sondez : Comment se rétablissent-ils soudainement ou progressivement ?*
- Après la fin de la maladie, un bébé a-t-il besoin de soins particuliers ? Quoi?
  - Pour combien de temps?
  - Où avez-vous appris cela ?
- Avez-vous déjà parlé à un membre de la communauté de ce qu'il faut faire pour un enfant lorsque sa maladie prend fin ?
  - Quand?
  - Qu'avez-vous dit?
  - Qu'est-ce qui vous a incité à donner ce conseil ?

**Alimentation pendant la maladie**

- Comment les enfants de 6 à 12 mois réagissent-ils aux aliments lorsqu'ils sont malades ?
  - *Sondez : Comment le bébé réagit-il à la nourriture au cours de différentes maladies ? Quelles sont les différences entre la diarrhée, la fièvre et la toux ?*
  - *Sondez : Comment réagit-il au lait maternel ? Certains aliments?*
  - *Sondez : Comment est son appétit ?*
  - Quelle quantité de nourriture mangent-ils lorsqu'ils sont malades, par rapport à la normale ?
    - *Sonder par type de maladie, gravité, âge si l'enfant*
  - Quelle quantité de lait maternel boivent-ils lorsqu'ils sont malades, par rapport à la normale ?
- Comment les bébés de cet âge devraient-ils manger ou boire lorsqu'ils sont malades ?
  - *Sondez : Que devraient-ils manger et boire ? Pourquoi?*
  - *Sondez : Quelle quantité de nourriture et de boisson faut-il leur donner ? Pourquoi?*
  - *Sondez : À quelle fréquence doivent-ils manger ? Pourquoi?*
  - Est-ce le même pour tous les types de maladies ?
- Quelles sont les raisons pour lesquelles les bébés ne mangent pas comme ils le devraient pendant la maladie ?
- Existe-t-il des aliments qui sont bons pour un bébé malade ?
  - Pourquoi sont-ils bons?
  - *Sondez : Est-ce le même pour tous les types de maladies ?*
- Y a-t-il des aliments qui sont mauvais pour un bébé malade ?
  - Pourquoi sont-ils mauvais?
  - *Sondez : Est-ce le même pour tous les types de maladies ?*
- Comment la maladie rend-elle l'alimentation d'un bébé plus facile ou plus difficile ?
  - *Sondez : Pour l'allaitement ? Pour l'alimentation d'autres aliments ?*
- Quels seraient les inconvénients de l'allaitement maternel plus fréquent en cas de maladie ? D'alimenter plus fréquemment d'autres aliments?
  - *Sondez : Aggrave la maladie, les tracas/le temps, augmente le coût de la nourriture*
- Quelle est la limite de la quantité de lait maternel que vous donnez à un bébé malade âgé de 6 à 12 mois ?
  - Qu'est-ce qui limite la quantité d'aliments solides ?
  - *Sonder : le temps de la mère, l'énergie de la mère, la production de lait, les conseils des autres, l'appétit du bébé*
  - *Sondez : Est-ce le même pour tous les types de maladies ?*
  - Que pouvez-vous faire pour inciter un enfant à manger davantage ? A boire plus de lait maternel?
- Y a-t-il des situations où vous pensez que vous ne devriez pas du tout allaiter votre bébé ?
  - *Sonder : selon le type de maladie, la gravité de la maladie, si la mère est elle-même malade*

**Nourrir après la maladie**

- Quelle quantité de nourriture un bébé de 6 à 12 mois doit-il consommer après une maladie ? (par rapport à avant la maladie)
  - Des sondes spécifiques:
    - Est-ce le même pour tous les types de maladies (par exemple, diarrhée, fièvre, toux) ? Si non, comment?
      - *Sondez : Aliments "chauds" et "froids" pour différentes maladies*
    - Est-ce la même chose pour les bébés filles et les bébés garçons ? Si non, comment?
    - Est-ce la même chose pour tous les repas ou seulement certains ? Si non, comment?
      - *Sondez : Comment l'alimentation après une maladie varie-t-elle au cours d'une journée ?*
    - Quand considérez-vous que c'est "après une maladie" ?
  - *S'ils disaient que le bébé devrait manger davantage après la maladie :*
    - Pourquoi?
    - Combien de temps cela devrait-il durer ?
    - Que faites-vous pour que le bébé mange davantage ? *(Sondez : préparez plus de nourriture, mettez plus de nourriture dans le bol d'alimentation, la même quantité dans le bol mais remplissez à nouveau le bol pendant l'alimentation si l'enfant a fini)*
    - La fréquence des repas doit-elle changer, ou la quantité de nourriture par repas ?
    - Y a-t-il des raisons pour lesquelles une famille pourrait ne pas nourrir le bébé davantage/plus de nourriture pendant quelques semaines après une maladie ?
  - *S'ils disaient que le bébé doit manger moins après la maladie :*
    - Pourquoi?
    - Combien de temps cela devrait-il durer ?
    - Que faites-vous pour que le bébé mange moins ? *(Sondez : préparez moins de nourriture, mettez moins de nourriture dans le bol d'alimentation, ne remplissez pas le bol pendant l'alimentation si l'enfant a fini)*
    - La fréquence des repas doit-elle changer, ou la quantité de nourriture par repas ?
  - *S'ils ont dit que le montant devrait être le même que d'habitude :*
    - Pourquoi ? *(Sondez : préférence de l'enfant, pas assez de nourriture ou de temps, l'enfant refuse, d'autres conseillent)*
- Ce que le bébé mange après une maladie doit-il être différent de ce qu'il mange normalement ?
  - Que sont les bons aliments ? Pourquoi?
  - Que sont les mauvais aliments ? Pourquoi?
  - *Sondez : Cela dépend-il du type de maladie ? Des préférences des enfants ? Du sexe de l'enfant ?*
  - Combien de temps cela devrait-il durer ?
  - Y a-t-il des raisons pour qu'une famille ne donne pas au bébé les aliments que vous avez mentionnés comme étant "bons" après une maladie ?
    - *Sondez : S'il n'y a pas assez de nourriture ? Pas assez de la bonne nourriture/favorite ? Pas assez de temps ? Si le bébé refuse de manger ? Un autre membre de la famille pourrait-il être en désaccord ?*
- Par rapport à la quantité de lait maternel qu'un bébé reçoit normalement, doit-il recevoir plus ou moins après une maladie ?
  - Est-ce que cela dépend du type de maladie ?
  - Est-ce la même chose pour les bébés filles et les bébés garçons ?
  - Combien de temps cela devrait-il durer ?
  - Y a-t-il des raisons pour lesquelles une mère ne ferait pas cela ?
    - *Sondez : Si la mère ne peut pas produire assez de lait, si le bébé refuse de manger, contraintes de temps, si quelqu'un n'est pas d'accord*
- Y a-t-il des familles qui donnent à leurs enfants des aliments supplémentaires pendant quelques semaines après la maladie ?
  - Qui ferait cela?
  - Pourquoi en donneraient-ils plus ?
  - Que penseraient ou diraient les autres ?
  - Que pensez-vous de ces familles ? (sont-elles anormales, pas comme moi, des modèles)

**Agence**

- Comment une famille peut-elle savoir si son enfant grandit bien ? *(sonde : poids/taille, forme du corps, énergie, comparaison avec les autres enfants)*
- Que peut faire une famille pour que son enfant grandisse bien ? *(Sondez : types de nourriture, quantité de nourriture, [autres ?])*
  - Qu'est-ce qui échappe au contrôle de la famille ? Pourquoi?
- Si un enfant ne grandit pas bien, à quoi cela ressemble-t-il ?
- Si un enfant tombe malade mais se rétablit plus tard, cela a-t-il un effet durable sur sa croissance ?
  - Quelle est la probabilité qu'elle ait un effet durable ?
  - Pourquoi la maladie a-t-elle parfois ces effets durables ? *(enquête : type ou quantité de nourriture que l'enfant consomme)*
  - Une famille peut-elle faire quelque chose pour s'assurer qu'elle continue à bien se développer après la maladie ? *(enquête : type de nourriture, quantité de nourriture ; enquête : pendant et après)*
    - Qu'est-ce qui échappe au contrôle de la famille ? Pourquoi?

**Interview Guide : Fathers and Grandmothers**

**Contexte familial**

- Pouvez-vous me parler un peu de vous ?
  - Quelle est votre journée type ? Votre âge?
- Pouvez-vous me parler de votre famille ?
  - *Sondez : Quels sont l'âge et le sexe des enfants ?*
  - Qui vit avec vous?
  - Quels sont les autres membres de la famille qui vivent à proximité ?
- Comment interagissez-vous avec le bébé au cours d'une semaine normale ?
  - À quelle fréquence interagissez-vous avec le bébé ?
  - Pendant combien de temps interagissez-vous ?

**Alimentation générale**

- Pourriez-vous décrire ce que votre famille mange au cours d'une journée typique ?
  - Comment cela pourrait-il changer en fonction du jour, de la semaine, du mois, de la saison ?
    - Quand peut-on se procurer davantage de nourriture ?
    - Quand y a-t-il moins de nourriture disponible ?
  - D'où provient votre nourriture (c'est-à-dire du marché, des stands le long des routes, de votre propre terrain, de vos voisins/ONG, collectée dans la nature)
    - Qui fait les courses ? / Qui collecte la nourriture ?
    - Quand font-ils leurs achats ou leurs collectes ?
  - Quand votre famille mange-t-elle ?
    - Combien de fois par jour la nourriture est-elle préparée ?
    - Combien de fois par jour les membres de la famille mangent-ils ?
  - Comment votre famille est-elle servie lors des repas ?
    - *Sondez : Lorsque votre famille prend un repas, dans quel ordre les gens mangent-ils ? Quand le bébé mange-t-il?*
- Qui décide quels aliments (le cas échéant) seront achetés ? Qui décide des aliments (le cas échéant) qui seront achetés ?
  - Qui décide du montant des dépenses consacrées à l'achat de nourriture pour la famille ?
    - *Si le père décide de l'argent mais que la mère fait les courses :* Quand obtenez-vous de l'argent pour les courses ? Recevez-vous le même montant chaque semaine ? Qu'est-ce que vous achetez ou fourrez habituellement ?
  - Qui prépare la nourriture lors d'une journée typique ?
- Que savez-vous sur ce que le bébé mange ?
  - Comment nourrir les enfants à l'âge de votre enfant (ou de 6 à 12 mois) ?
    - Vous trouvez quelque chose de difficile ? Intéressant?
- Pouvez-vous décrire tout ce que votre enfant a mangé hier ? (y compris l'allaitement)
  - Dans quelle mesure cela ressemble-t-il ou diffère-t-il d'une journée normale ?
  - Qui décide de ce que le bébé mange ?
  - Quel est l'aliment qui nourrit le plus votre bébé ?
    - *Sondez : Lait maternel, bouillie, fufu, ou autre aliment*
- Comment savoir si le bébé (à cet âge) mange bien ?
  - Comment savoir quand nourrir un bébé ?
  - Comment savoir quand il faut arrêter de nourrir un bébé ?
- Avez-vous donné à la mère des conseils ou des instructions sur la façon de nourrir un bébé ?
  - *Si oui :* Qu'avez-vous dit ?
  - *Si non:* pourquoi?
- Avez-vous déjà vu la mère encourager le bébé à manger davantage, alors qu'il semblait ne pas vouloir le faire ?
  - Qu'a-t-elle fait?
  - Que pensez-vous de cela ?
  - Y a-t-il quelque chose que les gens peuvent faire pour faire manger un bébé quand ils ne semblent pas intéressés ?

**Maladie**

- Dans cette communauté, quelle est la fréquence des maladies ?
  - Quel type de maladie?
  - Quelle saison?
  - Qui tombe généralement malade?
  - Que font les collectivités pour lutter contre ces maladies, le cas échéant ?
  - Si quelqu'un tombe malade, les personnes extérieures au foyer en entend-on généralement parler ?
  - Qui vous donne des conseils sur ce qu'il faut faire lorsqu'une personne tombe malade ?
  - Les gens mangent-ils généralement différemment pendant ou après la maladie ? Si oui, veuillez décrire comment.
- Est-il fréquent que les enfants de cette communauté tombent malades entre 6 mois et 1 an ?
  - Quelles sont les maladies les plus courantes ?
  - Quelles sont les causes de ces maladies ?
  - Certaines maladies sont-elles plus fréquentes à certaines périodes de l'année ? Lesquelles?
  - Que font les familles lorsque leur enfant est atteint de ces maladies ?
  - Qui donne des conseils sur ce qu'il faut faire lorsqu'un enfant tombe malade ?
- Comment savoir si votre bébé est malade ?
  - *Sondez : Sur la base des symptômes observés, modification des pleurs de l'enfant, modification de l'alimentation*
  - Différents pour différents enfants ? Différents selon l'âge de l'enfant ?
- Votre bébé est-il en bonne santé en ce moment ? *(N'oubliez pas : dans les guides de collecte de données, veillez à indiquer à l'enfant d'être orienté vers un établissement de santé spécifique et alertez le ReCo local)*
  - Comment le savez-vous?
- Qui est généralement la première personne à remarquer qu'un bébé de 6 à 12 mois est malade ? *(sonde pour chaque type de symptôme : diarrhée, toux, fièvre/corps chaud, ne pas manger)*
  - Comment les autres membres de la famille savent-ils si le bébé est malade ?
  - Si un enfant tombe malade, les personnes extérieures au foyer en entend-on parler ? Pourquoi ou pourquoi pas?
- Si vous remarquez que le bébé semble malade, que faites-vous ?
  - *Sondez : Est-ce qu'ils le diraient à leur mère, attendraient pour le dire à quelqu'un d'autre, attendraient pour le dire à quelqu'un ou ne feraient rien ?*
  - Pourquoi?
- Avez-vous donné à la mère des conseils ou des instructions sur ce qu'elle doit faire lorsque le bébé est malade ?
  - Quand et pourquoi?
  - Qu'avez-vous dit?
  - Dans votre famille, qui décide de ce qu'il faut faire lorsqu'un enfant de cet âge (6-12 mois) tombe malade ?
- Lorsque le bébé a *[demandez pour chacun : diarrhée, toux, fièvre/corps chaud]*, que fait votre famille ?
  - *Sonder : observer attentivement, rester à la maison, changer d'alimentation, demander conseil à des parents ou amis, aller dans un établissement de santé, guérisseur traditionnel, médecine, autres actions pour le confort physique*
- Que disent les gens de votre communauté au sujet du centre de santé local ? A propos des prestataires?
  - Que pensez-vous du centre de santé local ? A propos des prestataires?
  - Quel est le défi que représente une visite au centre de santé avec votre bébé ?

**Recouvrement**

- Lorsqu'un enfant de 6 à 12 mois est malade, combien de temps dure-t-il généralement ?
  - Comment savoir quand elle s'arrête ?
    - *Sondez : Si l'enfant pleure plus/moins, changement d'appétit de l'enfant, selon le fournisseur de soins, selon la mère*
    - *Sondez : Comment se rétablissent-ils soudainement ou progressivement ?*
- Après la fin de la maladie, un bébé a-t-il besoin de soins particuliers ? Quoi?
  - Pour combien de temps?

**Dernière maladie**

- Votre enfant est-il malade depuis l'âge de 6 mois ?
  - *Sonde : diarrhée, fièvre/corps chaud, ou toux ?*
  - Combien de fois votre bébé a-t-il été malade depuis l'âge de 6 mois ?
- Quand votre bébé a-t-il été malade pour la dernière fois depuis qu'il a atteint l'âge de 6 mois ?
  - Quel type de maladie a-t-il eu ?
  - Comment avez-vous su qu'il était malade ?
    - *Sondez : Symptômes observés, enfant refusé à la nourriture, changement dans les pleurs de l'enfant, quelqu'un leur a dit*
  - Pendant qu'il était malade, a-t-il continué à manger comme avant ?
    - *Si non :* qu'est-ce qui a changé ?
  - Qui s'est occupé de votre bébé pendant qu'il était malade ?
- Que faisait votre famille à l'époque où votre bébé était malade ?
  - *Sonder : se rendre au centre de santé, parler à ReCo, guérisseur traditionnel, donner des médicaments, surveiller quelque chose, changer d'alimentation (comment ?)*
  - Quand avez-vous fait cela ? (combien de temps après avoir remarqué qu'il était malade)
  - Qui a décidé de faire cela ?
- *(S'ils se sont rendus dans un établissement de santé ou ont parlé à la ReCo) :* Qui l'a amené dans l'établissement ou a contacté la ReCo ?
- Pendant combien de temps votre bébé a-t-il été malade ?
  - Comment avez-vous pu constater qu'il n'était plus malade ?
  - Lorsque vous avez pris soin de votre bébé juste après la maladie, votre famille a-t-elle fait quelque chose de différent de la façon dont vous vous occupez habituellement de lui ?

**Alimentation pendant la maladie**

- Depuis l'âge de 6 mois, comment votre bébé réagit-il aux aliments lorsqu'il est malade ?
  - *Sondez : Comment le bébé réagit-il à la nourriture au cours de différentes maladies ? Quelles sont les différences entre la diarrhée, la fièvre et la toux ?*
  - *Sondez : Comment réagit-il au lait maternel ? Certains aliments?*
  - *Sondez : Comment est son appétit ?*
  - Quelle quantité de nourriture votre bébé mange-t-il lorsqu'il est malade, par rapport à la normale ?
    - *Sonder par type de maladie, gravité, âge si l'enfant*
    - Comment le savez-vous?
  - Quelle quantité de lait maternel votre bébé boit-il lorsqu'il est malade, par rapport à la normale ?
    - Comment le savez-vous?
- Avez-vous déjà donné à la mère des conseils sur la façon de nourrir le bébé lorsqu'il était malade ?
  - Qu'avez-vous dit ?
  - Qu'est-ce qui vous a incité à donner des conseils ?
    - *Sondez : Les symptômes du bébé ? La réaction du bébé à la nourriture ?*
- Comment les bébés de cet âge devraient-ils manger ou boire lorsqu'ils sont malades ?
  - *Sondez : Que devraient-ils manger et boire ? Pourquoi?*
  - *Sondez : Quelle quantité de nourriture et de boisson faut-il leur donner ? Pourquoi?*
  - *Sondez : À quelle fréquence doivent-ils manger ? Pourquoi?*
  - Est-ce le même pour tous les types de maladies ?
- Quelles sont les raisons pour lesquelles les bébés ne mangent pas comme ils le devraient pendant la maladie ?
- Que doit faire la mère si le bébé n'a pas d'appétit ?
- Existe-t-il des aliments qui sont bons pour un bébé malade ?
  - Pourquoi sont-ils bons?
  - *Sondez : Est-ce le même pour tous les types de maladies ?*
- Y a-t-il des aliments qui sont mauvais pour un bébé malade ?
  - Pourquoi sont-ils mauvais?
  - *Sondez : Est-ce le même pour tous les types de maladies ?*
- Y a-t-il des situations où vous pensez qu'un enfant ne devrait pas du tout l'allaiter ?
  - *Sonder : selon le type de maladie, la gravité de la maladie, si la mère est elle-même malade*
- Comment les autres familles de votre communauté nourrissent-elles leurs bébés de 6 à 12 mois pendant la maladie ?
  - *Sondez : types d'aliments, quantité de nourriture, horaires d'alimentation*
  - Comment le savez-vous?
  - Est-ce le même pour tous les types de maladies ?
  - Est-ce que c'est la même chose pour tous les types de personnes dans la communauté ?

**Nourrir après la maladie**

- Quelle quantité de nourriture un bébé de 6 à 12 mois (ou l'âge de votre enfant) doit-il consommer après une maladie ? (par rapport à avant la maladie)
  - Des sondes spécifiques:
    - Est-ce le même pour tous les types de maladies (par exemple, diarrhée, fièvre, toux) ? Si non, comment?
      - *Sondez : Aliments "chauds" et "froids" pour différentes maladies*
    - Est-ce la même chose pour les bébés filles et les bébés garçons ? Si non, comment?
    - Est-ce la même chose pour tous les repas ou seulement certains ? Si non, comment?
      - *Sondez : Comment l'alimentation après une maladie varie-t-elle au cours d'une journée ?*
    - Quand considérez-vous que c'est "après une maladie" ?
  - *S'ils disaient que le bébé devrait manger davantage après la maladie :*
    - Pourquoi?
    - Combien de temps cela devrait-il durer ?
    - Que faites-vous pour que le bébé mange davantage ? *(Sondez : préparez plus de nourriture, mettez plus de nourriture dans le bol d'alimentation, la même quantité dans le bol mais remplissez à nouveau le bol pendant l'alimentation si l'enfant a fini)*
    - La fréquence des repas doit-elle changer, ou la quantité de nourriture par repas ?
    - Y a-t-il des raisons pour lesquelles une famille pourrait ne pas nourrir le bébé davantage/plus de nourriture pendant quelques semaines après une maladie ?
      - Est-ce que cela s'est déjà produit dans votre famille ? Racontez-moi l'histoire de cette époque.
  - *S'ils disaient que le bébé doit manger moins après la maladie :*
    - Pourquoi?
    - Combien de temps cela devrait-il durer ?
    - Que faites-vous pour que le bébé mange moins ? (*Sondez : préparez moins de nourriture, mettez moins de nourriture dans le bol d'alimentation, ne remplissez pas le bol pendant l'alimentation si l'enfant a fini)*
    - La fréquence des repas doit-elle changer, ou la quantité de nourriture par repas ?
  - *S'ils ont dit que le montant devrait être le même que d'habitude :*
    - Pourquoi ? *(Sondez : préférence de l'enfant, pas assez de nourriture ou de temps, l'enfant refuse, d'autres conseillent)*
- Ce que le bébé mange après une maladie doit-il être différent de ce qu'il mange normalement ?
  - Que sont les bons aliments ? Pourquoi ? Où avez-vous appris cela ?
  - Que sont les mauvais aliments ? Pourquoi ? Où avez-vous appris cela ?
  - *Sondez : Cela dépend-il du type de maladie ? Des préférences des enfants ? Du sexe de l'enfant ?*
  - Combien de temps cela devrait-il durer ?
  - Y a-t-il des raisons pour qu'une famille ne donne pas au bébé les aliments que vous avez mentionnés comme étant "bons" après une maladie ?
    - *Sondez : S'il n'y a pas assez de nourriture ? Pas assez de la bonne nourriture/favorite ? Pas assez de temps ? Si le bébé refuse de manger ? Un autre membre de la famille pourrait-il être en désaccord ?*
    - Est-ce que cela s'est déjà produit avec votre bébé ? Racontez-moi l'histoire de cette époque.
- Par rapport à la quantité de lait maternel qu'un bébé reçoit normalement, doit-il recevoir plus ou moins de lait après une maladie ?
  - Est-ce que cela dépend du type de maladie ?
  - Est-ce la même chose pour les bébés filles et les bébés garçons ?
  - Combien de temps cela devrait-il durer ?
  - Y a-t-il des raisons pour lesquelles une mère ne ferait pas cela ?
    - *Sondez : Si la mère ne peut pas produire assez de lait, si le bébé refuse de manger, contraintes de temps, si quelqu'un n'est pas d'accord*
    - Est-ce que cela s'est déjà produit avec votre bébé ? Racontez-moi l'histoire de cette époque.
- Comment savoir si quelque chose doit changer dans la façon dont le bébé est nourri pendant cette période ?
- Votre enfant mange-t-il généralement plus, moins ou la même quantité après une maladie ?
  - Pourquoi?
  - Comment savez-vous qu'il mange plus/moins ?
  - Comment cela évolue-t-il dans le temps (premiers jours, première semaine, deuxième semaine) ?
  - Que pensez-vous de l'idée de donner des aliments supplémentaires pendant cette période ? *(Sondez : quelles bonnes choses pourraient arriver à votre enfant et à votre famille en donnant au bébé de la nourriture supplémentaire ? Quelles sont les mauvaises choses qui pourraient se produire en donnant des aliments supplémentaires au bébé ?)*

**Influences**

- Comment les autres familles de votre communauté nourrissent-elles leurs bébés après une maladie ?
  - *Sondez : Quelle quantité de nourriture donnent-ils ? Quels aliments nourrissent-ils?*
  - Comment le savez-vous?
  - Que pensez-vous de cela ?
  - Que diriez-vous de donner à un enfant de la nourriture supplémentaire après une maladie?
- Y a-t-il des familles qui donnent à leurs enfants des aliments supplémentaires pendant quelques semaines après la maladie ?
  - Qui ferait cela?
  - Pourquoi en donneraient-ils plus ?
  - Que penseraient ou diraient les autres ?
  - Que pensez-vous de ces familles ? (sont-elles anormales, pas comme moi, des modèles)

**Agence**

- Comment une famille peut-elle savoir si son enfant grandit bien ? *(sonde : poids/taille, forme du corps, énergie, comparaison avec les autres enfants)*
- Que peut faire une famille pour que son enfant grandisse bien ? *(Sondez : types de nourriture, quantité de nourriture, [autres ?])*
  - Qu'est-ce qui échappe au contrôle de la famille ? Pourquoi?
- Si un enfant ne grandit pas bien, à quoi cela ressemble-t-il ?
- Si un enfant tombe malade mais se rétablit plus tard, cela a-t-il un effet durable sur sa croissance ?
  - Quelle est la probabilité qu'elle ait un effet durable ?
  - Pourquoi la maladie a-t-elle parfois ces effets durables ? *(enquête : type ou quantité de nourriture que l'enfant consomme)*
  - Une famille peut-elle faire quelque chose pour s'assurer qu'elle continue à bien se développer après la maladie ? *(enquête : type de nourriture, quantité de nourriture ; enquête : pendant et après)*
    - Qu'est-ce qui échappe au contrôle de la famille ? Pourquoi ?

**Interview Guide : Mothers**

**Contexte familial**

- Pouvez-vous me parler un peu de vous ?
  - Quelle est votre journée type ? Votre âge ?
- Pouvez-vous me parler de votre famille ?
  - *Sondez : Quels sont l'âge et le sexe des enfants ?*
  - Qui habite dans la maison ?
  - Vos parents ou votre belle-famille habitent-ils à proximité ? Une autre famille qui vit à proximité ?
- Qui interagit avec votre bébé au cours d'une semaine normale ?
  - *Sondez : les enfants plus âgés, le père, la grand-mère ? Autres membres de la famille ou voisins ?*
  - Que font-ils avec le bébé ? (c'est-à-dire regarder, jouer, nourrir, autres soins)
  - À quelle fréquence ?
- Travaillez-vous en dehors du domicile ?
  - *Si oui :* qui s'occupe de votre plus jeune enfant pendant cette période ? Pendant combien de temps à chaque fois ? À quelle distance se trouve l'endroit où vous travaillez ?
  - Quittez-vous la maison pour d'autres raisons sans le bébé (par exemple, pour vous rendre chez des parents, sur des marchés) ?

**Alimentation générale**

- Pourriez-vous décrire ce que votre famille mange au cours d'une journée typique ?
  - Comment cela pourrait-il changer en fonction du jour, de la semaine, du mois, de la saison ?
    - Quand peut-on se procurer davantage de nourriture ?
    - Quand y a-t-il moins de nourriture disponible ?
  - D'où provient votre nourriture (c'est-à-dire du marché, des stands le long des routes, de votre propre terrain, de vos voisins/ONG, collectée dans la nature)
    - Qui fait les courses ? / Qui collecte la nourriture ?
    - Quand font-ils leurs achats ou leurs collectes ?
  - Quand votre famille mange-t-elle ?
    - Combien de fois par jour préparez-vous de la nourriture ?
    - Combien de fois par jour les membres de la famille mangent-ils ?
  - Comment votre famille est-elle servie lors des repas ?
    - *Sondez : Lorsque votre famille prend un repas, dans quel ordre les gens mangent-ils ? Quand le bébé mange-t-il ?*
- Qui décide quels aliments (le cas échéant) seront achetés ? Qui décide des aliments (le cas échéant) qui seront achetés ?
  - Qui décide du montant des dépenses consacrées à l'achat de nourriture pour la famille ?
    - *Si le père décide de l'argent mais que la mère fait les courses :* Quand obtenez-vous de l'argent pour les courses ? Recevez-vous le même montant chaque semaine ? Qu'est-ce que vous achetez ou fourrez habituellement ?
  - Qui prépare la nourriture lors d'une journée typique ?
- Comment nourrir les enfants à l'âge de votre enfant (ou de 6 à 12 mois) ?
  - Vous trouvez quelque chose de difficile ? Intéressant ?
- Pouvez-vous décrire tout ce que votre enfant a mangé hier ? (y compris l'allaitement)
  - Que mangeait-il ?
  - Quand a-t-il mangé ? (Sondez : à quelle fréquence)
  - Combien a-t-il mangé à chaque fois ? *(demandez à utiliser vos mains pour démontrer la quantité)*
    - Comment savez-vous que c'est la quantité qu'il a mangée ?
  - Comment l'enfant a-t-il mangé ? (c'est-à-dire les doigts, le bol)
    - Qui a aidé l'enfant à manger, le cas échéant ?
- Dans quelle mesure cela ressemble-t-il ou diffère-t-il d'une journée normale ?
  - Quels autres aliments votre bébé mange-t-il occasionnellement ? À quelle fréquence mange-t-il ces aliments ?
  - Quels sont les aliments préférés de votre enfant ? Comment pouvez-vous le savoir ?
  - Qui décide de ce que le bébé mange ?
  - Quel est l'aliment qui nourrit le plus votre bébé ?
    - *Sondez : Lait maternel, bouillie, fufu, ou autre aliment*
- Comment savoir si votre bébé (à cet âge) mange bien ?
  - Y a-t-il des jours où votre enfant a un gros appétit par rapport à d'autres jours ou d'autres moments ? Quand est-ce le cas ?
  - Votre bébé (à cet âge) refuse-t-il parfois la nourriture que vous lui offrez ? Quels signes ? Que faites-vous dans ce cas ?
  - Quand décidez-vous de nourrir votre bébé ?
    - *Sondez : Comment savoir quand il faut allaiter ? Quand nourrir d'autres aliments ?*
  - Quand décidez-vous d'arrêter de nourrir votre bébé ?
    - *Sondez : Comment savoir quand il faut arrêter d'allaiter ? Quand arrêter de donner d'autres aliments ?*
  - Vous avez une carte de croissance pour votre bébé ? Si oui, pouvons-nous la voir ?
    - *[Prendre des photos de la carte]*
    - Avez-vous déjà utilisé cette carte ? Comment ?
- Quand les bébés ne semblent-ils pas intéressés par la nourriture ou les liquides ?
  - Y a-t-il quelque chose que les gens peuvent faire pour faire manger un bébé quand ils ne semblent pas intéressés ?
    - Y a-t-il des trucs *que vous* avez essayé de faire manger au bébé, mais celui-ci a continué à refuser la nourriture ?
  - Avez-vous déjà vu quelqu'un cajoler ou encourager un bébé à manger alors qu'il n'en avait pas envie ?
    - Que faisaient-ils ?
    - Qu'en avez-vous pensé ?
  - Avez-vous déjà vu ou entendu parler de quelqu'un qui forçait un bébé à manger alors qu'il ne le voulait pas ?
    - Que faisaient-ils ?
    - Que pensez-vous de cela ?

**Maladie**

- Dans cette communauté, quelle est la fréquence des maladies ?
  - Quel type de maladie ?
  - Quelle saison ?
  - Qui tombe généralement malade ?
  - Que font les collectivités pour lutter contre ces maladies, le cas échéant ?
  - Si quelqu'un tombe malade, les personnes extérieures au foyer en entend-on généralement parler ?
  - Qui vous donne des conseils sur ce qu'il faut faire lorsqu'une personne tombe malade ?
  - Les gens mangent-ils généralement différemment pendant ou après la maladie ? Si oui, veuillez décrire comment.
- Est-il fréquent que les enfants de cette communauté tombent malades entre 6 mois et 1 an ?
  - Quelles sont les maladies les plus courantes ?
  - Quelles sont les causes de ces maladies ?
  - Certaines maladies sont-elles plus fréquentes à certaines périodes de l'année ? Lesquelles ?
  - Que font les familles lorsque leur enfant est atteint de ces maladies ?
  - Qui donne des conseils sur ce qu'il faut faire lorsqu'un enfant tombe malade ?
- Comment savoir si votre bébé est malade ?
  - *Sondez : Sur la base des symptômes observés, modification des pleurs de l'enfant, modification de l'alimentation*
  - Différents pour différents enfants ? Différents selon l'âge de l'enfant ?
- Votre bébé est-il en bonne santé en ce moment ? *(N'oubliez pas : dans les guides de collecte de données, veillez à indiquer à l'enfant d'être orienté vers un établissement de santé spécifique et alertez le ReCo local)*
  - Comment le savez-vous ?
- Qui est généralement la première personne à remarquer qu'un bébé de 6 à 12 mois est malade ? *(sonde pour chaque type de symptôme : diarrhée, toux, fièvre/corps chaud, ne pas manger)*
  - Comment les autres membres de la famille savent-ils si le bébé est malade ?
  - Quand informeriez-vous les autres membres de la famille des symptômes d'un bébé ?
  - Dans quelles circonstances n'informeriez-vous pas les autres membres de la famille des symptômes d'un bébé ?
  - Si un enfant tombe malade, les personnes extérieures au foyer en entend-on parler ? Pourquoi ou pourquoi pas ?
- Lorsque votre enfant a *[demandez pour chacun : diarrhée, toux, fièvre/corps chaud]*, que faites-vous ?
  - *Sonder : observer attentivement, rester à la maison, changer d'alimentation, demander conseil à des parents ou amis, aller dans un établissement de santé, guérisseur traditionnel, médecine, autres actions pour le confort physique*
  - Comment vous sentez-vous ?
  - Où avez-vous appris à faire cela ?
- Que disent les gens de votre communauté au sujet du centre de santé local ? A propos des prestataires ?
  - Que pensez-vous du centre de santé local ? A propos des prestataires ?
  - Quel est le défi de visiter le centre de santé avec votre bébé ?
- Dans votre famille, qui décide de ce qu'il faut faire lorsqu'un enfant de cet âge (6-12 mois) tombe malade ?
- Lorsque le bébé est malade (avec diarrhée, avec toux, avec corps chaud/ fièvre), votre vie quotidienne change-t-elle ? Si oui, comment ? Que faites-vous et que font les autres différemment, le cas échéant ?

**Recouvrement**

- Lorsqu'un enfant de 6 à 12 mois est malade, combien de temps dure-t-il généralement ?
  - Comment savoir quand elle s'arrête ?
    - *Sondez : Si l'enfant pleure plus/moins, changement d'appétit de l'enfant, selon le fournisseur de soins, selon la mère*
    - *Sondez : Comment se rétablissent-ils soudainement ou progressivement ?*
  - Qui, dans votre famille, est le premier à remarquer que le bébé se rétablit ?
  - A qui feriez-vous confiance pour savoir si votre bébé est toujours malade ou non ?
- Lorsqu'un bébé se rétablit, votre vie quotidienne change-t-elle ? Si oui, comment ?
- Après la fin de la maladie, un bébé a-t-il besoin de soins particuliers ? Quoi ?
  - Pour combien de temps ?
  - Où avez-vous appris cela ?

**Dernière maladie**

- Votre enfant est-il malade depuis l'âge de 6 mois ?
  - *Sonde : diarrhée, fièvre/corps chaud, ou toux ?*
  - Combien de fois votre bébé a-t-il été malade depuis l'âge de 6 mois ?
- Quand votre bébé a-t-il été malade pour la dernière fois depuis qu'il a atteint l'âge de 6 mois ?
  - Quel type de maladie a-t-il eu ?
  - Comment avez-vous su qu'il était malade ?
    - *Sondez : Symptômes observés, enfant refusé à la nourriture, changement dans les pleurs de l'enfant, quelqu'un leur a dit*
  - Qui d'autre savait que votre bébé était malade ?
    - Comment l'ont-ils su ?
  - Pendant qu'il était malade, a-t-il continué à manger comme avant ?
    - *Si non :* qu'est-ce qui a changé ?
  - Qui s'est occupé de votre bébé pendant qu'il était malade ?
- Qu'avez-vous fait à l'époque où votre bébé était malade ?
  - *Sonder : se rendre au centre de santé, parler à ReCo, guérisseur traditionnel, donner des médicaments, surveiller quelque chose, changer d'alimentation (comment ?)*
  - Comment avez-vous su faire cela ? Qui vous a conseillé ?
  - Quand avez-vous fait cela ? (combien de temps après avoir remarqué qu'il était malade)
  - Qui a décidé de faire cela ?
- *(S'ils se sont rendus dans un établissement de santé ou ont parlé à la ReCo) :* Qui l'a amené dans l'établissement ou a contacté la ReCo ?
  - Qu'a fait et dit le prestataire/ReCo lorsque vous les avez vus ?
    - *Sondez : Vous ont-ils donné des conseils sur les aliments ou les liquides à donner à l'enfant ?*
  - Qu'avez-vous pensé de ce conseil ? *(Sondez : dans quelle mesure vous a-t-il été possible de suivre ces conseils chez vous ?)*
- Pendant combien de temps votre bébé a-t-il été malade ?
  - Comment avez-vous pu constater qu'il n'était plus malade ?
  - Lorsque vous vous êtes occupée de votre bébé juste après la maladie, avez-vous fait quelque chose de différent de la façon dont vous vous occupez habituellement de lui ?

**Alimentation pendant la maladie**

- Depuis l'âge de 6 mois, comment votre bébé réagit-il aux aliments lorsqu'il est malade ?
  - *Sondez : Comment le bébé réagit-il à la nourriture au cours de différentes maladies ? Quelles sont les différences entre la diarrhée, la fièvre et la toux ?*
  - *Sondez : Comment réagit-il au lait maternel ? Certains aliments ?*
  - *Sondez : Comment est son appétit ?*
  - Quelle quantité de nourriture votre bébé mange-t-il lorsqu'il est malade, par rapport à la normale ?
    - *Sondez par type de maladie, gravité, âge si l'enfant*
    - Comment le savez-vous ?
  - Quelle quantité de lait maternel votre bébé boit-il lorsqu'il est malade, par rapport à la normale ?
    - Comment le savez-vous ?
  - Vous avez parlé de la façon dont on peut persuader un bébé de manger alors qu'il ne semble pas intéressé. La dernière fois que votre bébé a été malade, avez-vous essayé des trucs - lesquels, et comment votre bébé a-t-il réagi ?
- Comment les bébés de cet âge devraient-ils manger ou boire lorsqu'ils sont malades ?
  - *Sondez : Que devraient-ils manger et boire ? Pourquoi ?*
  - *Sondez : Quelle quantité de nourriture et de boisson faut-il leur donner ? Pourquoi ?*
  - *Sondez : À quelle fréquence doivent-ils manger ? Pourquoi ?*
  - Est-ce le même pour tous les types de maladies ?
  - Où avez-vous appris cela ?
- Quelles sont les raisons pour lesquelles les bébés ne mangent pas comme ils le devraient pendant la maladie ? Comment vous sentez-vous si votre bébé ne mange pas comme il le devrait lorsqu'il est malade ?
- Si un bébé ne mange pas comme il le devrait pendant une maladie, qui dans le ménage le remarque ? Qui d'autre en dehors du ménage le remarque ? Que disent-ils ?
- Existe-t-il des aliments qui sont bons pour un bébé malade ?
  - Pourquoi sont-ils bons ?
  - Où avez-vous appris cela ?
  - *Sondez : Est-ce le même pour tous les types de maladies ?*
- Y a-t-il des aliments qui sont mauvais pour un bébé malade ?
  - Pourquoi sont-ils mauvais ?
  - Comment le savez-vous ?
  - *Sondez : Est-ce le même pour tous les types de maladies ?*
- Depuis que votre bébé a 6 mois, avez-vous déjà changé ce que vous lui donniez à manger lorsqu'il était malade ?
  - Qu'est-ce qui a motivé ce changement ?
    - *Sonde : en raison de l'aggravation des symptômes, la réaction du bébé à la nourriture, d'autres conseillent*
- Comment la maladie rend-elle l'alimentation de votre bébé plus facile ou plus difficile ?
  - *Sondez : Pour l'allaitement ? Pour l'alimentation d'autres aliments ?*
- Quels seraient les inconvénients de l'allaitement maternel plus fréquent en cas de maladie ? D'alimenter plus fréquemment d'autres aliments ?
  - *Sondez : Aggrave la maladie, les tracas/le temps, augmente le coût de la nourriture*
- Quelle est la limite de la quantité de lait maternel que vous donnez à un bébé malade âgé de 6 à 12 mois ?
  - Qu'est-ce qui limite la quantité d'aliments solides ?
  - *Sonder : le temps de la mère, l'énergie de la mère, la production de lait, les conseils des autres, l'appétit du bébé*
  - *Sondez : Est-ce le même pour tous les types de maladies ?*
- Y a-t-il des situations où vous pensez que vous ne devriez pas du tout allaiter votre bébé ?
  - *Sonder : selon le type de maladie, la gravité de la maladie, si la mère est elle-même malade*

**Influences**

- Comment votre propre mère vous a-t-elle nourri lorsque vous étiez malade ?
- Comment les autres familles de votre communauté nourrissent-elles leurs bébés de 6 à 12 mois pendant la maladie ?
  - *Sondez : types d'aliments, quantité de nourriture, horaires d'alimentation*
  - Comment le savez-vous ?
  - Est-ce le même pour tous les types de maladies ?
  - Est-ce que c'est la même chose pour tous les types de personnes dans la communauté ?
- La façon dont vous nourrissez les bébés malades a-t-elle changé depuis que votre premier enfant était un bébé ? Comment ? Pourquoi ?
- Un professionnel de la santé ou un agent de santé communautaire vous a-t-il déjà dit comment nourrir votre bébé ?
  - Qu'ont-ils dit sur l'alimentation d'un enfant en général ?
  - Qu'ont-ils dit sur l'alimentation d'un enfant malade ?

**Alimentation après la maladie**

- Quelle quantité de nourriture un bébé de 6 à 12 mois (ou l'âge de votre enfant) doit-il consommer après une maladie ? (par rapport à avant la maladie)
  - Des sondes spécifiques :
    - Est-ce le même pour tous les types de maladies (par exemple, diarrhée, fièvre, toux) ? Si non, comment ?
      - *Sondez : Aliments "chauds" et "froids" pour différentes maladies*
    - Est-ce la même chose pour les bébés filles et les bébés garçons ? Si non, comment ?
    - Est-ce la même chose pour tous les repas ou seulement certains ? Si non, comment ?
      - *Sondez : Comment l'alimentation après une maladie varie-t-elle au cours d'une journée ?*
    - Quand considérez-vous que c'est "après une maladie" ?
    - Comment la présence d'enfants plus âgés affecte-t-elle l'alimentation de votre plus jeune après la maladie ?
  - *S'ils disaient que le bébé devrait manger davantage après la maladie :*
    - Pourquoi ?
    - Combien de temps cela devrait-il durer ?
    - Que faites-vous pour que le bébé mange davantage ? *(Sondez : préparez plus de nourriture, mettez plus de nourriture dans le bol d'alimentation, la même quantité dans le bol mais remplissez à nouveau le bol pendant l'alimentation si l'enfant a fini)*
    - La fréquence des repas doit-elle changer, ou la quantité de nourriture par repas ?
    - Y a-t-il des raisons pour lesquelles une famille pourrait ne pas nourrir le bébé davantage/plus de nourriture pendant quelques semaines après une maladie ?
      - Est-ce que cela s'est déjà produit dans votre famille ? Racontez-moi l'histoire de cette époque.
  - *S'ils disaient que le bébé doit manger moins après la maladie :*
    - Pourquoi ?
    - Combien de temps cela devrait-il durer ?
    - Que faites-vous pour que le bébé mange moins ? *(Sondez : préparez moins de nourriture, mettez moins de nourriture dans le bol d'alimentation, ne remplissez pas le bol pendant l'alimentation si l'enfant a fini)*
    - La fréquence des repas doit-elle changer, ou la quantité de nourriture par repas ?
  - *S'ils ont dit que le montant devrait être le même que d'habitude :*
    - Pourquoi ? *(Sondez : préférence de l'enfant, pas assez de nourriture ou de temps, l'enfant refuse, d'autres conseillent)*
- Ce que le bébé mange après une maladie doit-il être différent de ce qu'il mange normalement ?
  - Que sont les bons aliments ? Pourquoi ? Où avez-vous appris cela ?
  - Que sont les mauvais aliments ? Pourquoi ? Où avez-vous appris cela ?
  - *Sondez : Cela dépend-il du type de maladie ? Des préférences des enfants ? Du sexe de l'enfant ?*
  - Combien de temps cela devrait-il durer ?
  - Y a-t-il des raisons pour qu'une famille ne donne pas au bébé les aliments que vous avez mentionnés comme étant "bons" après une maladie ?
    - *Sondez : S'il n'y a pas assez de nourriture ? Pas assez de la bonne nourriture/favorite ? Pas assez de temps ? Si le bébé refuse de manger ? Un autre membre de la famille pourrait-il être en désaccord ?*
    - Est-ce que cela s'est déjà produit avec votre bébé ? Racontez-moi l'histoire de cette époque.
- Par rapport à la quantité de lait maternel qu'un bébé reçoit normalement, doit-il recevoir plus ou moins de lait après une maladie ?
  - Est-ce que cela dépend du type de maladie ?
  - Est-ce la même chose pour les bébés filles et les bébés garçons ?
  - Combien de temps cela devrait-il durer ?
  - Y a-t-il des raisons pour lesquelles une mère ne ferait pas cela ?
    - *Sondez : Si la mère ne peut pas produire assez de lait, si le bébé refuse de manger, contraintes de temps, si quelqu'un n'est pas d'accord*
    - Est-ce que cela s'est déjà produit avec votre bébé ? Racontez-moi l'histoire de cette époque.
- Comment savoir si quelque chose doit changer dans la façon dont le bébé est nourri pendant cette période ?
- Votre enfant mange-t-il généralement plus, moins ou la même quantité après une maladie ?
  - Pourquoi ?
  - Comment savez-vous qu'il mange plus/moins ?
  - Comment cela évolue-t-il dans le temps (premiers jours, première semaine, deuxième semaine) ?
  - Que pensez-vous de l'idée de donner des aliments supplémentaires pendant cette période ? *(Sondez : quelles bonnes choses pourraient arriver à votre enfant et à votre famille en donnant au bébé de la nourriture supplémentaire ? Quelles sont les mauvaises choses qui pourraient se produire en donnant des aliments supplémentaires au bébé ?)*

**Influences**

- Comment les autres familles de votre communauté nourrissent-elles leurs bébés après une maladie ?
  - *Sondez : Quelle quantité de nourriture donnent-ils ? Quels aliments nourrissent-ils ?*
  - Comment le savez-vous ?
  - Que pensez-vous de cela ?
  - Que diriez-vous de donner à un enfant de la nourriture supplémentaire après une maladie ?
- Y a-t-il des familles qui donnent à leurs enfants des aliments supplémentaires pendant quelques semaines après la maladie ?
  - Qui ferait cela ?
  - Pourquoi en donneraient-ils plus ?
  - Que penseraient ou diraient les autres ?
  - Que pensez-vous de ces familles ? (sont-elles anormales, pas comme moi, des modèles)

**Agence**

- Comment une famille peut-elle savoir si son enfant grandit bien ? *(sonde : poids/taille, forme du corps, énergie, comparaison avec les autres enfants)*
- Que peut faire une famille pour que son enfant grandisse bien ? *(Sondez : types de nourriture, quantité de nourriture, [autres ?])*
  - Qu'est-ce qui échappe au contrôle de la famille ? Pourquoi ?
- Si un enfant ne grandit pas bien, à quoi cela ressemble-t-il ?
- Si un enfant tombe malade mais se rétablit plus tard, cela a-t-il un effet durable sur sa croissance ?
  - Quelle est la probabilité qu'elle ait un effet durable ?
  - Pourquoi la maladie a-t-elle parfois ces effets durables ? *(enquête : type ou quantité de nourriture que l'enfant consomme)*
  - Une famille peut-elle faire quelque chose pour s'assurer qu'elle continue à bien se développer après la maladie ? *(enquête : type de nourriture, quantité de nourriture ; enquête : pendant et après)*

**Interview Guide : Health Providers**

**Rôle**

- **Quel est votre rôle dans cette installation et quelles sont vos principales responsabilités ?**
  - Combien de prestataires y a-t-il dans cet établissement ? Quelles sont leurs responsabilités?
    - Sonder : combien de personnes : médecins, infirmières, sages-femmes, internes ?
  - À quoi ressemble une bonne journée à l'établissement ? Qu'est-ce qu'une mauvaise journée ?

**Services**

- **Pour quels problèmes de santé les personnes s'occupant d'un enfant de moins d'un an se rendent-elles généralement dans un centre de santé ?**
  - Qui est généralement la personne qui amène l'enfant au centre de santé ?
    - Combien de fois les pères / LMI accompagnent-ils la mère ?
  - Quelles sont les campagnes de santé qui se déroulent actuellement dans la communauté ?
  - Combien de ReCos votre centre de santé ou votre communauté possède-t-il ?
    - Quelles sont les actions de ReCos dans la communauté ?
  - Que font généralement les mères lorsqu'un enfant souffre de toux, de diarrhée ou de fièvre ?
    - Où les mères vont-elles généralement en premier ?
      - *Sondez :* Des centres de santé ? Guérisseurs traditionnels ? ReCos ?
    - Combien de temps les mères attendent-elles généralement avant de demander des soins pour leur enfant malade ?
    - Selon vous, quels indices les mères utilisent-elles pour décider du moment où elles doivent se faire soigner (Sondez le nombre de jours de maladie, la gravité des symptômes, la fréquence/la rareté de la maladie chez les enfants dans la communauté)
- **Que se passe-t-il lors d'une visite de vaccination (CPS) ? Veuillez me guider du début à la fin.**
  - Quels types de mères ont accès à ces services ? Quels sont les types de mères qui n'y ont pas accès ?
    - Combien de mères sur 10 dans cette communauté estimez-vous qu'elles visitent le centre de vaccination ?
    - Les mères qui viennent à une campagne/événement spécial de vaccination sont-ils différents de ceux qui viennent à l'établissement un jour de routine pour les vaccinations ? De quelle manière ?
  - Sur quels sujets, le cas échéant, conseillez-vous les mères lors de cette visite ?
    - Quel est le sujet le plus important dont vous parlez aux mères ?
    - Quels conseils donnez-vous lors des journées de vaccination ? L'interaction que vous avez avec les soignants est-elle différente ces jours-là par rapport à une visite typique dans un centre de santé ?
- **Que se passe-t-il lors de la visite d'un enfant malade au centre de santé pour un enfant de moins d'un an ? Veuillez me guider du début à la fin.**
  - Quand existe-t-il des services pour la santé des enfants ?
    - Sondez : Certains jours ? Certaines heures ?
    - Quels sont les prestataires qui offrent ces services ?
      - Sondez : médecins, infirmières, sages-femmes, autres ?
  - Quelles sont les raisons typiques pour lesquelles une mère fait appel à ce service ? Quels sont les symptômes de son bébé?
    - *Sondez :* Fièvre, diarrhée, toux, perte de poids
  - Quels types de mères amènent leurs bébés malades à ce centre de santé ? Quels types de mères n'y vont pas?
    - Combien de mères sur 10 dans cette communauté estimez-vous qu'elles se rendent dans l'établissement pour y chercher des soins pour leur enfant malade ?
    - Quelles sont les questions que vous posez à la mère sur l'état de l'enfant ?
    - Quel type de conseil donnez-vous aux mères ? Est-il différent en fonction de l'enfant (sexe, âge, vaccination ou autres antécédents médicaux) ? En fonction de la mère ? Si oui, comment/qu'est-ce que vous dites à chaque groupe ?
    - Comment savoir ce qu'il est important de dire à une mère lorsqu'elle amène un enfant malade ?
  - Dites-vous aux mères comment savoir quand la maladie est terminée ? Que leur dites-vous?
    - Quels sont les défis, s'il y en a, que vous avez à relever pour parler de ce sujet ?
    - Comment savoir si les mères comprennent bien ?
- **Quels sujets, le cas échéant, conseillez-vous aux mères lors d'une visite pour diarrhée ?**
  - Quel est le sujet le plus important dont vous parlez aux mères ?
    - Que conseillez-vous aux mères ?
    - Sondez : Cela diffère-t-il selon que l'enfant a moins ou plus de 6 mois ? Si oui, en quoi les conseils diffèrent-ils ?
  - Environ combien de temps passez-vous avec une mère lors d'une visite pour diarrhée ?
  - Parlez-vous de nutrition au cours de cet entretien ? Si oui, qu'en dites-vous ?
  - Quel est le rôle de l'alimentation dans le traitement des enfants et l'aide à la guérison de la diarrhée ?
- **Quels sujets, le cas échéant, conseillez-vous aux mères lors d'une visite pour fièvre ?**
  - Quel est le sujet le plus important dont vous parlez aux mères ?
    - Que conseillez-vous aux mères ?
    - Sondez : Cela diffère-t-il selon que l'enfant a moins ou plus de 6 mois ? Si oui, en quoi les conseils diffèrent-ils ?
  - Environ combien de temps passez-vous avec une mère pendant une visite pour cause de fièvre ?
  - Parlez-vous de nutrition au cours de cet entretien ? Si oui, qu'en dites-vous ? Quel est le rôle de l'alimentation pour soigner les enfants et les aider à se remettre de la fièvre ?
- **Quels sujets, le cas échéant, conseillez-vous aux mères lors d'une visite pour tousser ?**
  - Quel est le sujet le plus important dont vous parlez aux mères ?
    - Que conseillez-vous aux mères ?
    - Sondez : Cela diffère-t-il selon que l'enfant a moins ou plus de 6 mois ? Si oui, en quoi les conseils diffèrent-ils ?
  - Environ combien de temps passez-vous avec une mère pendant une visite de toux ?
  - Parlez-vous de nutrition au cours de cet entretien ? Si oui, qu'en dites-vous ? Quel est le rôle de l'alimentation dans le traitement des enfants et l'aide à la guérison de la toux ?

**Nutrition**

1. **Comment les mères de cette communauté nourrissent-elles leurs enfants entre 6 et 12 mois ?**
   - À quel âge les enfants cessent-ils généralement d'allaiter ici ?
     - Sur 10 mères, combien allaitent leur enfant...
       - pour les 12 premiers mois ?
       - pendant les 24 premiers mois ?
   - Quels sont les produits alimentaires que les bébés mangent entre 6 et 12 mois ?
     - *Sonde : manioc, manioc, riz, viande, yaourt, œufs*
     - Quand les mères introduisent-elles ces différents aliments ?
       - Comment les aliments fournis au bébé changent-ils de 6 à 12 mois ? De 12 à 24 mois ?
   - Qui décide de ce que le bébé doit manger à cet âge ?
     - La mère reçoit-elle des conseils de quelqu'un d'autre ?
     - Quel est le rôle du mari ? Belle-mère ? Quelqu'un d'autre ?
   - Comment décririez-vous l'état nutritionnel des enfants âgés de 6 à 12 mois dans cette communauté ? Et l'état nutritionnel des enfants de moins de 2 ans ?
   - Comment pensez-vous que l'alimentation pendant les 6 à 12 mois affecte la croissance d'un enfant pendant ses premières années de vie ?
   - Qui est responsable de veiller à ce qu'un enfant reçoive une alimentation suffisante ? Pourquoi ?
     - *Sonder :* mère, mari, grand-mère/MIL, ReCo, vulgarisateurs agricoles, agents de santé
   - Quand discutez-vous de la nutrition avec les mères ?
     - *Sonder :* campagne de santé, CPN, CPS, visite d'un enfant malade - pour quelles maladies ?
     - De quoi parlez-vous ?

**Alimentation de récupération *pendant la* maladie**

1. **La plupart des agents de santé conseillent-ils les mères sur l'alimentation pendant la maladie ?**
   - Si non:
     - Quelles sont les raisons pour lesquelles les agents de santé ne discutent pas de l'alimentation pendant la maladie lors de ces visites ?
       - *Sondez : le* temps presse, d'autres sujets sont plus critiques, l'enfant est trop malade, les mères le savent déjà ?
   - Si oui:
     - Que diriez-vous à une mère de nourrir son enfant pendant sa maladie ?
       - *Sondez :* Avez-vous autre chose à dire ? Sur la fréquence, la quantité, le type d'aliments ?
     - Utiliserez-vous des aides ou leur fournirez-vous du matériel ?
     - Comment la mère réagit-elle à ce conseil ?
     - Quelles questions, le cas échéant, les femmes posent-elles généralement ?
       - *Sondez :* Quelles sont leurs préoccupations, le cas échéant ?
     - Selon vous, qu'est-ce que la mère apprend de la séance de conseil ? Qu'est-ce qu'elle n'apprend pas?
       - Pensez-vous que les mères suivent les conseils des agents de santé concernant l'alimentation de l'enfant pendant la maladie ?
         - S'ils le font, qu'est-ce qui les aide à le faire ?
         - S'ils ne le font pas, pourquoi ?
2. **Quels aliments ou liquides une mère doit-elle offrir à son enfant de 6 à 12 mois pendant sa maladie ? Pourquoi ?**
   - Quel est l'aliment / le liquide le plus important à offrir à un enfant malade pendant sa maladie ? Pourquoi ?
     - *Sonde : lait maternel, bouillie, eau, autres aliments*
   - Quels sont les aliments/liquides qu'elle *ne* doit *pas* proposer ? Pourquoi ?
   - Que conseilleriez-vous à la mère si l'enfant refuse la nourriture ou le lait maternel ?
   - Cela varie-t-il en fonction de la maladie ? Quels sont les autres facteurs qui influencent l'alimentation de l'enfant ?
3. **Comment une mère doit-elle nourrir son enfant de 6 à 12 mois pendant la maladie ?**
   - À quelle fréquence la mère doit-elle nourrir l'enfant ? Quelle quantité doit-elle lui donner à manger ?
     - Y a-t-il quelque chose qui limite cela ? *Sondez : l'appétit, le coût - même si on lui donnait les connaissances nécessaires, qu'est-ce qui lui rend difficile de se nourrir de cette façon lorsque l'enfant est malade ? Quand est-il plus facile pour les mères de nourrir un enfant malade comme vous le dites ?*
   - Comment doit-elle préparer la nourriture (par rapport à une alimentation normale) ?
   - Comment doit-elle nourrir l'enfant ?
     - Quelles techniques la mère peut-elle utiliser pour encourager l'alimentation ?
     - *Sondez :* Si un enfant ne mange pas, la mère doit-elle le forcer à manger ?
   - Que ne doit *pas* faire une mère concernant l'alimentation d'un enfant pendant la maladie ?

**Alimentation de récupération *après une* maladie**

1. **Quels sont les signes qui indiquent qu'un bébé a "récupéré" d'une maladie ?**
   - Quand une maladie se "termine" ? Comment une mère peut-elle savoir que la maladie est terminée ?
     - *Sondez :* Quels sont les signes de la diarrhée ? De la fièvre ? De la toux ?
       - *Sonder :* symptômes, appétit, médicaments de finition
     - Comment les femmes apprennent-elles les signes de fin de maladie, si elles le font ?
       - Avez-vous l'habitude de parler de ce sujet aux mères lors de la visite d'un enfant malade ? Que leur dites-vous ?
   - Après la fin de la maladie, un enfant de 6 à 12 mois a-t-il besoin de soins particuliers ?
     - Y a-t-il une période de récupération ? Quelle est sa durée ?
     - Que doit faire une mère pendant cette période ?
       - Avez-vous l'habitude d'en parler aux mères lors de la visite d'un enfant malade ? Qu'en dites-vous ?
       - Qui d'autre la conseille ou la soutient ? (Sondez le suivi après la visite de l'enfant malade, la visite de la ReCo, les membres du ménage, les membres de la famille)
   - La mère ramène-t-elle parfois son enfant de 6 à 12 mois au centre de santé après la fin de la maladie, lorsqu'il commence à se rétablir ? Quels sont les types de mères qui ramènent leur enfant après la maladie ? Si oui, quels sont les types de mères qui ramènent leur enfant après la maladie ?
     - Que se passe-t-il lors de cette visite ?
     - Lors de cette visite, les prestataires conseillent-ils à la mère de donner des aliments supplémentaires à l'enfant de 6 à 12 mois pendant sa convalescence ? Que disent-ils généralement ? Si ce n'est pas le cas, pourquoi ?
     - Quel est le pourcentage de mères de votre communauté qui donnent de la nourriture supplémentaire après une maladie ? Pendant combien de temps ? Selon vous, qu'est-ce qui rend difficile ou facile pour une mère de donner de la nourriture supplémentaire ? (coût, saison, soutien familial, nombre d'enfants)
2. **Quels aliments ou liquides une mère doit-elle offrir à son enfant de 6 à 12 mois pendant les quelques semaines qui *suivent* une maladie, lorsque l'enfant se rétablit ?**
   - Pourquoi ces aliments ?
   - En quelles quantités doit-elle offrir des aliments après la maladie ?
   - En quoi cela diffère-t-il des aliments/liquides qu'elle doit offrir *pendant la* maladie ?
   - Quelle est la nourriture / le liquide le plus important à offrir à un enfant en convalescence après une maladie ?
   - Quels sont les aliments/liquides qu'elle *ne* doit *pas* proposer ? Pourquoi ?
   - Que conseilleriez-vous à la mère si l'enfant refuse la nourriture ou le lait maternel ?
     - Le manque d'appétit après une maladie est-il fréquent ?
3. **Comment une mère doit-elle nourrir son enfant de 6 à 12 mois *après la* maladie ?**
   - En quoi cela diffère-t-il de la manière dont elle doit fournir des aliments/liquides *pendant la* maladie ?
     - Dans quelle mesure l'enfant a-t-il de l'appétit *après une* maladie ?
   - À quelle fréquence la mère doit-elle nourrir l'enfant ? Quelle quantité de nourriture supplémentaire la mère doit-elle donner ? Pendant combien de temps ?
   - Comment doit-elle préparer la nourriture (par rapport à une alimentation normale) ?
   - Comment doit-elle inciter l'enfant à manger ?
   - Quelles techniques la mère peut-elle utiliser pour encourager l'alimentation ?
     - Dans quelle mesure l'encouragement est-il nécessaire *après la* maladie ?
   - Que conseilleriez-vous à la mère de *ne pas* faire ?
   - Sur dix, combien de mères de votre communauté se nourrissent en plus après que leur bébé ait été malade ?

**Expérience des prestataires de soins de santé en matière d'alimentation de récupération**

1. Comment un prestataire peut-il savoir si un enfant se développe bien ? *(sonde : poids/taille, forme du corps, énergie, comparaison avec les autres enfants)*
   - Que peut faire un prestataire pour assurer la bonne croissance d'un enfant ? *(enquête : types de nourriture, quantité de nourriture, [autres ?])*
     - Qu'est-ce qui échappe au contrôle du prestataire ? Pourquoi ?
   - Si un enfant ne grandit pas bien, à quoi cela ressemble-t-il ?
2. Si un enfant tombe malade mais se rétablit plus tard, cela a-t-il un effet durable sur sa croissance ?
   - Quelle est la probabilité qu'elle ait un effet durable ?
   - Pourquoi la maladie a-t-elle parfois ces effets durables ? *(enquête : type ou quantité de nourriture que l'enfant consomme)*
   - Un prestataire peut-il faire quelque chose pour garantir que l'enfant continue à bien grandir après la maladie ? *(enquête : type de nourriture, quantité de nourriture ; enquête : pendant et après)*
     - Qu'est-ce qui échappe au contrôle du prestataire ? Pourquoi ?
3. **Comment les performances sont-elles évaluées pour des prestataires comme vous ?**
   - Comment savez-vous si vous vous en sortez bien dans votre rôle ?
   - Quel type de retour d'information le personnel de cette clinique reçoit-il ? Qui donne ce retour d'information ?
4. Où trouvez-vous des informations sur la croissance des nourrissons (taille, poids) ?
   - Comment les performances des prestataires sont-elles évaluées en fonction de ces résultats ?
5. **Les résultats liés à l'alimentation des nourrissons sont-ils suivis dans cet établissement ? Les résultats liés aux maladies infantiles sont-ils suivis ?**
   - Y a-t-il un registre ici ? Puis-je prendre une photo ? *[Assurez-vous que la photo ne comporte pas d'informations d'identification personnelle, c'est-à-dire toute information qui pourrait permettre d'identifier une personne, notamment son nom, son adresse, son numéro de téléphone et son numéro de carte d'identité].*
   - Comment les performances des prestataires sont-elles évaluées en fonction de ces résultats ?
6. **Quels types de formations les prestataires de cet établissement reçoivent-ils ?**
   - Avez-vous reçu des formations sur les maladies infantiles ? Sur la nutrition des enfants ?
     - L'une de ces formations mentionnait-elle l'alimentation pendant ou après la maladie ?
     - [*Si oui*] Quand a eu lieu la dernière formation ? De quoi s'agissait-il ?
7. **Des aides à l'emploi ou des outils sont-ils utilisés pour vous aider à conseiller les enfants malades lors de leurs visites ?**
   - *[Demandez à voir ces outils, et prenez des photos de ceux-ci].*
   - Pourriez-vous me montrer comment vous utiliseriez cet outil pour conseiller la mère ?
8. **Utilisez-vous des aides à l'emploi ou des outils pour vous aider dans vos conseils sur la nutrition des enfants ?**
   - *[Demandez à voir ces outils, et prenez des photos de ceux-ci].*
   - Pourriez-vous me montrer comment vous utiliseriez cet outil pour conseiller la mère ?

**Interview Guide : Community Health Workers**

**Rôle**

1. **Quel est votre rôle en tant que ReCo dans cette communauté et quelles sont vos principales responsabilités ?**
   - Combien y a-t-il de ReCos dans cette communauté ? Quelles sont leurs responsabilités ?
     - Sonder : combien de personnes : médecins, infirmières, sages-femmes, internes ?
   - À quoi ressemble une bonne journée à l'établissement ? Qu'est-ce qu'une mauvaise journée ?

**Services**

1. **Pour quels problèmes de santé les personnes s'occupant d'un enfant de moins d'un an se rendent-elles généralement dans un centre de santé ?**
   - Qui est généralement la personne qui amène l'enfant au centre de santé ?
     - Combien de fois les pères / LMI accompagnent-ils la mère ?
   - Quelles sont les campagnes de santé qui se déroulent actuellement dans la communauté ?
   - Combien de ReCos votre centre de santé ou votre communauté possède-t-il ?
     - Quelles sont les actions de ReCos dans la communauté ?
   - Que font généralement les mères lorsqu'un enfant souffre de toux, de diarrhée ou de fièvre ?
     - Où les mères vont-elles généralement en premier ?
       - *Sondez :* Des centres de santé ? Guérisseurs traditionnels ? ReCos ?
     - Combien de temps les mères attendent-elles généralement avant de demander des soins pour leur enfant malade ?
     - Selon vous, quels indices les mères utilisent-elles pour décider du moment où elles doivent se faire soigner (Sondez le nombre de jours de maladie, la gravité des symptômes, la fréquence/la rareté de la maladie chez les enfants dans la communauté)
2. **Que se passe-t-il lors d'une campagne de vaccination ? Veuillez me guider du début à la fin.**
   - Quels types de mères ont accès à ces services ? Quels sont les types de mères qui n'y ont pas accès ?
     - Combien de mères sur 10 dans cette communauté estimez-vous qu'elles visitent le centre de vaccination ?
     - Les mères qui viennent à une campagne/événement spécial de vaccination sont-ils différents de ceux qui viennent à l'établissement un jour de routine pour les vaccinations ? De quelle manière ?
   - Sur quels sujets, le cas échéant, conseillez-vous les mères lors de cette visite ?
     - `Quel est le sujet le plus important dont vous parlez aux mères ?
     - Quels conseils donnez-vous lors des journées de vaccination ? L'interaction que vous avez avec les soignants est-elle différente ces jours-là par rapport à une visite typique dans un centre de santé ?
3. **Que se passe-t-il lors de la visite d'un enfant malade au centre de santé pour un enfant de moins d'un an ? Veuillez me guider du début à la fin.**
   - Quand existe-t-il des services pour la santé des enfants ?
     - Sondez : Certains jours ? Certaines heures ?
     - Quels sont les ReCos / fournisseurs qui offrent ces services ?
       - Sondez : médecins, infirmières, sages-femmes, autres ?
   - Quelles sont les raisons typiques pour lesquelles une mère fait appel à ce service ? Quels sont les symptômes de son bébé ?
     - *Sondez :* Fièvre, diarrhée, toux, perte de poids
   - Pour quelles raisons une mère s'adresserait-elle à une ReCo pour obtenir de l'aide pour un enfant malade ?
     - Quels types de mères amènent leurs bébés malades à une ReCo ? Quels types de mères ne le font pas ?
     - Selon vous, combien de mères sur dix dans cette communauté s'adresseraient à une ReCo pour obtenir des soins pour leur enfant malade ?
     - Quelles sont les questions que vous posez à la mère sur l'état de l'enfant ?
     - Quel type de conseil donnez-vous aux mères ? Est-il différent en fonction de l'enfant (sexe, âge, vaccination ou autres antécédents médicaux) ? En fonction de la mère ? Si oui, comment/qu'est-ce que vous dites à chaque groupe ?
     - Comment savoir ce qu'il est important de dire à une mère lorsqu'elle amène un enfant malade ?
   - Dites-vous aux mères comment savoir quand la maladie est terminée ? Que leur dites-vous?
     - Quels sont les défis, s'il y en a, que vous avez à relever pour parler de ce sujet ?
     - Comment savoir si les mères comprennent bien ?
4. **Quels sujets, le cas échéant, conseillez-vous aux mères pour la diarrhée ?**
   - Quel est le sujet le plus important dont vous parlez aux mères ?
     - Que conseillez-vous aux mères ?
     - Sondez : Cela diffère-t-il selon que l'enfant a moins ou plus de 6 mois ? Si oui, en quoi les conseils diffèrent-ils?
   - Environ combien de temps passez-vous avec une mère pendant un entretien sur la diarrhée ?
   - Parlez-vous de nutrition au cours de cet entretien ? Si oui, qu'en dites-vous ?
   - Quel est le rôle de l'alimentation dans le traitement des enfants et l'aide à la guérison de la diarrhée ?
5. **Quels sujets, le cas échéant, conseillez-vous aux mères pour la fièvre ?**
   - Quel est le sujet le plus important dont vous parlez aux mères ?
     - Que conseillez-vous aux mères ?
     - Sondez : Cela diffère-t-il selon que l'enfant a moins ou plus de 6 mois ? Si oui, en quoi les conseils diffèrent-ils?
   - Combien de temps passez-vous avec une mère lors d'un entretien sur la fièvre ?
   - Parlez-vous de nutrition au cours de cet entretien ? Si oui, qu'en dites-vous ? Quel est le rôle de l'alimentation pour soigner les enfants et les aider à se remettre de la fièvre ?
6. **Quels sujets, le cas échéant, conseillez-vous aux mères pour la toux ?**
   - Quel est le sujet le plus important dont vous parlez aux mères ?
     - Que conseillez-vous aux mères ?
     - Sondez : Cela diffère-t-il selon que l'enfant a moins ou plus de 6 mois ? Si oui, en quoi les conseils diffèrent-ils?
   - Environ combien de temps passez-vous avec une mère pendant un entretien de toux ?
   - Parlez-vous de nutrition au cours de cet entretien ? Si oui, qu'en dites-vous ? Quel est le rôle de l'alimentation dans le traitement des enfants et l'aide à la guérison de la toux ?

**Nutrition**

1. **Comment les mères de cette communauté nourrissent-elles leurs enfants entre 6 et 12 mois ?**
   - À quel âge les enfants cessent-ils généralement d'allaiter ici ?
     - Sur 10 mères, combien allaitent leur enfant...
       - pour les 12 premiers mois ?
       - pendant les 24 premiers mois ?
   - Quels sont les produits alimentaires que les bébés mangent entre 6 et 12 mois ?
     - *Sonde : manioc, manioc, riz, viande, yaourt, œufs*
     - Quand les mères introduisent-elles ces différents aliments ?
       - Comment les aliments fournis au bébé changent-ils de 6 à 12 mois ? De 12 à 24 mois ?
   - Qui décide de ce que le bébé doit manger à cet âge ?
     - La mère reçoit-elle des conseils de quelqu'un d'autre ?
     - Quel est le rôle du mari ? Belle-mère ? Quelqu'un d'autre ?
   - Comment décririez-vous l'état nutritionnel des enfants âgés de 6 à 12 mois dans cette communauté ? Et l'état nutritionnel des enfants de moins de 2 ans ?
   - Comment pensez-vous que l'alimentation pendant les 6 à 12 mois affecte la croissance d'un enfant pendant ses premières années de vie ?
   - Qui est responsable de veiller à ce qu'un enfant reçoive une alimentation suffisante ? Pourquoi ?
     - *Sonder :* mère, mari, grand-mère/MIL, ReCo, vulgarisateurs agricoles, agents de santé
   - Quand est-ce que vous ou un prestataire de soins en établissement discutez de la nutrition avec les mères ?
     - *Sonder :* campagne de santé, CPN, CPS, visite d'un enfant malade - pour quelles maladies ?
     - De quoi parlez-vous ?

**Alimentation de récupération *pendant la* maladie**

1. **La plupart des agents de santé conseillent-ils les mères sur l'alimentation pendant la maladie ?**
   - Si non :
     - Quelles sont les raisons pour lesquelles les agents de santé ne discutent pas de l'alimentation pendant la maladie lors de ces visites ?
       - *Sondez : le* temps presse, d'autres sujets sont plus critiques, l'enfant est trop malade, les mères le savent déjà ?
   - Si oui :
     - Que diriez-vous à une mère de nourrir son enfant pendant sa maladie ?
       - *Sondez :* Avez-vous autre chose à dire ? Sur la fréquence, la quantité, le type d'aliments ?
     - Utiliserez-vous des aides ou leur fournirez-vous du matériel ?
     - Comment la mère réagit-elle à ce conseil ?
     - Quelles questions, le cas échéant, les femmes posent-elles généralement ?
       - *Sondez :* Quelles sont leurs préoccupations, le cas échéant ?
     - Selon vous, qu'est-ce que la mère apprend de la séance de conseil ? Qu'est-ce qu'elle n'apprend pas?
       - Pensez-vous que les mères suivent les conseils des agents de santé concernant l'alimentation de l'enfant pendant la maladie ?
         - S'ils le font, qu'est-ce qui les aide à le faire ?
         - S'ils ne le font pas, pourquoi ?
2. **Quels aliments ou liquides une mère doit-elle offrir à son enfant de 6 à 12 mois pendant sa maladie ? Pourquoi ?**
   - Quel est l'aliment / le liquide le plus important à offrir à un enfant malade pendant sa maladie ? Pourquoi ?
     - *Sonde : lait maternel, bouillie, eau, autres aliments*
   - Quels sont les aliments/liquides qu'elle *ne* doit *pas* proposer ? Pourquoi ?
   - Que conseilleriez-vous à la mère si l'enfant refuse la nourriture ou le lait maternel ?
   - Cela varie-t-il en fonction de la maladie ? Quels sont les autres facteurs qui influencent l'alimentation de l'enfant ?
3. **Comment une mère doit-elle nourrir son enfant de 6 à 12 mois pendant la maladie ?**
   - À quelle fréquence la mère doit-elle nourrir l'enfant ? Quelle quantité doit-elle lui donner à manger ?
     - Y a-t-il quelque chose qui limite cela ? *Sondez : l'appétit, le coût - même si on lui donnait les connaissances nécessaires, qu'est-ce qui lui rend difficile de se nourrir de cette façon lorsque l'enfant est malade ? Quand est-il plus facile pour les mères de nourrir un enfant malade comme vous le dites ?*
   - Comment doit-elle préparer la nourriture (par rapport à une alimentation normale) ?
   - Comment doit-elle nourrir l'enfant ?
     - Quelles techniques la mère peut-elle utiliser pour encourager l'alimentation ?
     - *Sondez :* Si un enfant ne mange pas, la mère doit-elle le forcer à manger ?
   - Que ne doit *pas* faire une mère concernant l'alimentation d'un enfant pendant la maladie ?

**Alimentation de récupération *après une* maladie**

1. **Quels sont les signes qui indiquent qu'un bébé a "récupéré" d'une maladie ?**
   - Quand une maladie se "termine" ? Comment une mère peut-elle savoir que la maladie est terminée ?
     - *Sondez :* Quels sont les signes de la diarrhée ? De la fièvre ? De la toux ?
       - *Sonder :* symptômes, appétit, médicaments de finition
     - Comment les femmes apprennent-elles les signes de fin de maladie, si elles le font ?
       - Avez-vous l'habitude de parler de ce sujet aux mères lors de la visite d'un enfant malade ? Que leur dites-vous ?
   - Après la fin de la maladie, un enfant de 6 à 12 mois a-t-il besoin de soins particuliers ?
     - Y a-t-il une période de récupération ? Quelle est sa durée ?
     - Que doit faire une mère pendant cette période ?
       - Avez-vous l'habitude d'en parler aux mères lors de la visite d'un enfant malade ? Que leur dites-vous ?
       - Qui d'autre la conseille ou la soutient ? (Sondez le suivi après la visite de l'enfant malade, la visite de la ReCo, les membres du ménage, les membres de la famille)
   - La mère ramène-t-elle parfois son enfant de 6 à 12 mois à la ReCo / au centre de santé après la fin de la maladie, lorsqu'il commence à se rétablir ? Si oui :
     - Quels sont les types de mères qui ramènent leur enfant à la ReCo / au centre de santé après une maladie ?
     - Que se passe-t-il lors de cette visite ?
     - Lors de cette visite, les prestataires ReCos conseillent-ils à la mère de donner des aliments supplémentaires à l'enfant de 6 à 12 mois pendant sa convalescence ?
       - Que disent-ils habituellement ? Si ce n'est pas le cas, pourquoi ?
     - Quel est le pourcentage de mères de votre communauté qui donnent de la nourriture supplémentaire après une maladie ? Pendant combien de temps ? Selon vous, qu'est-ce qui rend difficile ou facile pour une mère de donner de la nourriture supplémentaire ? (coût, saison, soutien familial, nombre d'enfants)
2. **Quels aliments ou liquides une mère doit-elle offrir à son enfant de 6 à 12 mois pendant les quelques semaines qui *suivent* une maladie, lorsque l'enfant se rétablit ?**
   - Pourquoi ces aliments?
   - En quelles quantités doit-elle offrir des aliments après la maladie ?
   - En quoi cela diffère-t-il des aliments/liquides qu'elle doit offrir *pendant la* maladie ?
   - Quelle est la nourriture / le liquide le plus important à offrir à un enfant en convalescence après une maladie ?
   - Quels sont les aliments/liquides qu'elle *ne* doit *pas* proposer ? Pourquoi?
   - Que conseilleriez-vous à la mère si l'enfant refuse la nourriture ou le lait maternel ?
     - Le manque d'appétit après une maladie est-il fréquent ?
3. **Comment une mère doit-elle nourrir son enfant de 6 à 12 mois *après la* maladie ?**
   - En quoi cela diffère-t-il de la manière dont elle doit fournir des aliments/liquides *pendant la* maladie ?
     - Dans quelle mesure l'enfant a-t-il de l'appétit *après une* maladie ?
   - À quelle fréquence la mère doit-elle nourrir l'enfant ? Quelle quantité de nourriture supplémentaire la mère doit-elle donner ? Pendant combien de temps?
   - Comment doit-elle préparer la nourriture (par rapport à une alimentation normale) ?
   - Comment doit-elle inciter l'enfant à manger ?
   - Quelles techniques la mère peut-elle utiliser pour encourager l'alimentation ?
     - Dans quelle mesure l'encouragement est-il nécessaire *après la* maladie ?
   - Que conseilleriez-vous à la mère de *ne pas* faire ?
   - Sur dix, combien de mères de votre communauté se nourrissent en plus après que leur bébé ait été malade ?

**L'expérience du ReCo en matière d'alimentation de récupération**

1. Comment une ReCo peut-elle savoir si un enfant se développe bien ? *(sonde : poids/taille, forme du corps, énergie, comparaison avec les autres enfants)*
   - Que peut faire une ReCo pour assurer la bonne croissance d'un enfant ? *(enquête : types de nourriture, quantité de nourriture, [autres ?])*
     - Qu'est-ce qui échappe au contrôle de la ReCo ? Pourquoi ?
   - Si un enfant ne grandit pas bien, à quoi cela ressemble-t-il ?
2. Si un enfant tombe malade mais se rétablit plus tard, cela a-t-il un effet durable sur sa croissance ?
   - Quelle est la probabilité qu'elle ait un effet durable ?
   - Pourquoi la maladie a-t-elle parfois ces effets durables ? *(enquête : type ou quantité de nourriture que l'enfant consomme)*
   - Une ReCo peut-elle faire quelque chose pour s'assurer que l'enfant continue à bien grandir après la maladie ? *(enquête : type de nourriture, quantité de nourriture ; enquête : pendant et après)*
     - Qu'est-ce qui échappe au contrôle de la ReCo ? Pourquoi ?
3. **Comment sont évaluées les performances de ReCos comme vous ?**
   - Comment savez-vous si vous vous en sortez bien dans votre rôle ?
   - Quels sont les commentaires que ReCos reçoit dans cette communauté ? Qui donne ce feedback ?
4. **Où trouvez-vous des informations sur la croissance des nourrissons (taille, poids) ?**
   - Comment les performances de ReCos sont-elles évaluées en fonction de ces résultats ?
5. **ReCos suit-il les résultats liés à l'alimentation des nourrissons ? Y a-t-il des résultats liés aux maladies infantiles qui font l'objet d'un suivi ?**
   - Avez-vous un registre à cet effet ? Puis-je prendre une photo ? *[Assurez-vous que la photo ne comporte pas d'informations d'identification personnelle, c'est-à-dire toute information qui pourrait permettre d'identifier une personne, notamment son nom, son adresse, son numéro de téléphone et son numéro de carte d'identité].*
   - Comment les performances de ReCos sont-elles évaluées en fonction de ces résultats ?
6. **Quels types de formations ReCos reçoit-elle dans cet établissement ?**
   - Avez-vous reçu des formations sur les maladies infantiles ? Sur la nutrition des enfants ?
     - L'une de ces formations mentionnait-elle l'alimentation pendant ou après la maladie ?
     - [*Si oui*] Quand a eu lieu la dernière formation ? De quoi s'agissait-il ?
7. **Des aides à l'emploi ou des outils sont-ils utilisés pour vous aider à conseiller les enfants malades lors de leurs visites ?**
   - *[Demandez à voir ces outils, et prenez des photos de ceux-ci].*
   - Pourriez-vous me montrer comment vous utiliseriez cet outil pour conseiller la mère ?
8. **Utilisez-vous des outils ou des aides à l'emploi pour vous conseiller sur la nutrition des enfants ?**
   - *[Demandez à voir ces outils, et prenez des photos de ceux-ci].*
   - Pourriez-vous me montrer comment vous utiliseriez cet outil pour conseiller la mère ?

**Observation Guide : General Visit**

**Chercheur : Date :**

**Centre de santé : Province/District :**

**Lieu : Urbain / Périurbain / Rural**

## Aperçu

1. Décrivez brièvement la famille qui est venue pour cette visite
   1. Quel est l'âge approximatif de l'enfant ? _________
   2. Qui est avec eux ? (Encercler toutes les réponses) Mère Grand-mère Père Autres enfants
   3. L'enfant semble-t-il être en bonne ou en mauvaise santé ? Décrivez tous les symptômes que vous pouvez observer, notamment si l'enfant est pâle, s'il vomit, s'il est faible ou s'il tousse.

## Contexte d’une visite de Santé Générale (encerclez un: CPON / CPS)

1. Heure de début : _____________
2. Où a lieu la consultation ? Décrivez l'espace de consultation.
3. Quelqu'un d'autre peut-il voir ou entendre la consultation ? Y a-t-il des distractions ou des interruptions?
4. Cochez les sujets qui sont abordés pendant la session. Si oui, décrivez ce qui a été couvert dans l'espace à droite de chaque élément.

- Nutrition
  - La relation entre les aliments nutritifs et la vie/ résultats en matière de santé
  - Diversité alimentaire
  - Signes d'une bonne alimentation chez un enfant
  - Mesure de croissance / pesée
- Allaitement maternel exclusif
- Alimentation complémentaire
  - Fréquence d'alimentation + invites à se nourrir
  - Quantité de denrées alimentaires à nourrir
- Maladie
  - Signes de déshydratation
  - Signes et symptômes de la maladie
  - Recherche de soins pour les maladies infantiles
  - Importance de l'alimentation pendant la maladie
  - Importance de l'alimentation pendant la *convalescence/rétablissement*
  - Traitement des maladies (y compris les SOR)
- Alimentation pendant la maladie
  - Combien/quelle fréquence d'allaitement d'un enfant pendant la maladie
  - Quelle quantité, quelle fréquence et quels aliments donner à un enfant pendant la maladie
  - Quelle quantité/combien de fois/quels liquides nourrir un enfant pendant la maladie
- Alimentation après la maladie
  - Combien/quelle fréquence d'allaitement d'un enfant après une maladie
  - Quelle quantité, quelle fréquence et quels aliments donner à un enfant après une maladie
  - Quelle quantité/combien de fois/quels liquides nourrir un enfant après une maladie
- Autres :

1. Le prestataire dit-il quelque chose sur l'état nutritionnel actuel de l'enfant ? Décrivez ce que dit le prestataire et comment la famille réagit.
2. Le prestataire utilise-t-il des outils médicaux ou du matériel de communication pendant la consultation, ou donne-t-il quelque chose à la famille pour qu'elle l'emporte chez elle ?
3. Décrivez ce qui est utilisé et comment.
4. Décrivez comment le prestataire explique la ressource.
5. Si du outil médical ou du matériel de communication est donné à la famille, décrivez ce qu'elle en fait dans la salle de consultation.
6. Après la fin de la consultation, prenez des photos d’une copie propre du matériel utilisé (ne pas prendre de photo des informations du client).
7. Décrivez la disposition de la famille pendant la consultation.
   1. La famille semble-t-elle prêter attention au prestataire ? Quel est le langage corporel du membre de la famille par rapport au prestataire?
   2. Réagissent-ils très positivement ou très négativement à tout ce que dit le prestataire ? Si oui, décrivez à quoi ils réagissent et comment ils réagissent.
   3. Notez s'ils semblent nerveux, irrités, distraits, intimidés ou confus. Notez quelles étaient les circonstances immédiates qui ont conduit à cette réaction.
   4. Notez toutes les questions posées par la famille.
8. Décrivez la disposition du prestataire pendant la consultation. Notez s'ils semblent pressés, inquiets, irrités, distraits ou joyeux.
9. Le prestataire communique-t-il à la famille les points clés à retenir et les étapes suivantes ? Décrivez-les brièvement.
10. Décrivez la réaction de la famille aux conclusions et aux prochaines étapes, ainsi que les réactions des prestataires à la famille.
11. Heure de clôture: _______________

**Observation Guide : Sick Visit**

**Chercheur : Date :**

**Centre de santé : Province/District :**

**Lieu : Urbain / Périurbain / Rural**

## Contexte d’une visite d’un enfant malade

1. Heure de début : _____________
2. Décrivez brièvement la famille qui est venue pour cette visite
   1. Quel est l'âge approximatif de l'enfant ? _________
   2. Qui est avec eux ? (Encercler toutes les réponses) Mère Grand-mère Père Autres enfants
   3. L'enfant semble-t-il être en bonne ou en mauvaise santé ? Décrivez tous les symptômes que vous pouvez observer, notamment si l'enfant est pâle, s'il vomit, s'il est faible ou s'il tousse.
3. Où a lieu la consultation ? Décrivez l'espace de consultation.
4. Quelqu'un d'autre peut-il voir ou entendre la consultation ? Y a-t-il des distractions ou des interruptions?
5. Décrivez la disposition de la famille pendant la consultation.
   1. La famille semble-t-elle prêter attention au prestataire ? Quel est le langage corporel du membre de la famille par rapport au prestataire?
   2. Réagissent-ils très positivement ou très négativement à tout ce que dit le prestataire ? Si oui, décrivez à quoi ils réagissent et comment ils réagissent.
   3. Notez s'ils semblent nerveux, irrités, distraits, intimidés ou confus. Notez quelles étaient les circonstances immédiates qui ont conduit à cette réaction.
   4. Notez toutes les questions posées par la famille.
6. Décrivez la disposition du prestataire pendant la consultation. Notez s'ils semblent pressés, inquiets, irrités, distraits ou joyeux.
7. Le prestataire utilise-t-il des outils médicaux ou du matériel de communication pendant la consultation, ou donne-t-il quelque chose à la famille pour qu'elle l'emporte chez elle ?
8. Décrivez ce qui est utilisé et comment.
9. Décrivez comment le prestataire explique la ressource.
10. Si du outil médical ou du matériel de communication est donné à la famille, décrivez ce qu'elle en fait dans la salle de consultation.
11. Après la fin de la consultation, prenez des photos d’une copie propre du matériel utilisé (ne pas prendre de photo des informations du client).

## Contenu de la discussion

1. Cochez les sujets qui sont abordés pendant la session. Si oui, décrivez ce qui a été couvert dans l'espace à droite de chaque élément.

- Nutrition
  - La relation entre les aliments nutritifs et la vie/ résultats en matière de santé
  - Diversité alimentaire
  - Signes d'une bonne alimentation chez un enfant
  - Mesure de croissance / pesée
- Allaitement maternel exclusif
- Alimentation complémentaire
  - Fréquence d'alimentation + invites à se nourrir
  - Quantité de denrées alimentaires à nourrir
- Maladie
  - Signes de déshydratation
  - Signes et symptômes de la maladie
  - Recherche de soins pour les maladies infantiles
  - Importance de l'alimentation pendant la maladie
  - Importance de l'alimentation pendant la *convalescence/rétablissement*
  - Traitement des maladies (y compris les SOR)
- Alimentation pendant la maladie
  - Combien/quelle fréquence d'allaitement d'un enfant pendant la maladie
  - Quelle quantité, quelle fréquence et quels aliments donner à un enfant pendant la maladie
  - Quelle quantité/combien de fois/quels liquides nourrir un enfant pendant la maladie
- Alimentation après la maladie
  - Combien/quelle fréquence d'allaitement d'un enfant après une maladie
  - Quelle quantité, quelle fréquence et quels aliments donner à un enfant après une maladie
  - Quelle quantité/combien de fois/quels liquides nourrir un enfant après une maladie
- Autres :

1. Le prestataire mentionne-t-il quelque chose à propos de la poursuite de l'alimentation pendant la maladie ?
   1. Si oui, décrivez leurs conseils.
   2. Sur quoi le prestataire met-il l'accent ? (par exemple, l'allaitement, les liquides, l'alimentation a base d’ aliments mous, l'hygiène) ?
   3. Le prestataire mentionne-t-il quelque chose que l'enfant ne doit PAS manger ou boire pendant sa maladie ? Si oui, quoi ?
   4. Le prestataire mentionne-t-il la quantité à manger ou ce qu'il faut manger pendant la maladie ?
   5. Décrivez comment la famille réagit à ces conseils. Semblent-ils attentifs et comprennent-ils ce que le prestataire a dit ? Notez toutes les questions qu'ils posent.
   6. Combien de temps (en minutes) dure la discussion sur l'alimentation pendant la maladie ?
2. Le prestataire mentionne-t-il des stratégies pour encourager/cajoleries un enfant malade à manger/ boire/ allaiter ?
   1. Si oui, quelles sont les stratégies qu'elle mentionne ?
   2. Utilise-t-elle des ressources ? (photos, poupées, livrets, etc.) Si oui, décrivez.
   3. Fait-elle des démonstrations? Si oui, décrivez.
   4. Comment le parent réagit-il à cette partie de la consultation ?
3. Décrivez tout ce que le prestataire dit sur la durée de la maladie ou sur la façon dont la famille saura que l'enfant a commencé à se rétablir.
   1. Quels changements, le cas échéant, suggèrent-ils d'apporter lorsque l'enfant a commencé à se rétablir ?
   2. Offrent-ils des conseils sur la manière de savoir quand un enfant est *complètement rétabli* ? Si oui, que disent-ils ?
4. Le prestataire mentionne-t-il quelque chose sur l'alimentation après la maladie ?
   1. Si oui, décrivez leurs conseils.
   2. Sur quoi le prestataire met-il l'accent ? (par exemple, l'allaitement, les liquides, l'alimentation en aliments mous, l'ajout d'un repas supplémentaire) ?
   3. Le prestataire mentionne-t-il la quantité à manger ou ce qu'il faut manger après la maladie ?
   4. Décrivez comment la famille réagit à ces conseils. Semblent-ils attentifs et comprennent-ils ce que le prestataire a dit ? Notez toutes les questions qu'ils posent.
   5. Combien de temps (en minutes) dure la discussion sur l'alimentation après la maladie ?
5. Le prestataire dit-il quelque chose sur l'état nutritionnel actuel de l'enfant ? Décrivez ce que dit la personne qui s'occupe de l'enfant et comment la famille réagit.

## Prochaines Étapes

1. Le prestataire communique-t-il à la famille les points clés à retenir et les étapes suivantes ? Décrivez-les brièvement.
2. Décrivez la réaction de la famille aux conclusions et aux prochaines étapes, ainsi que les réactions des prestataires à la famille.
3. Si le prestataire recommande un traitement :
   1. Quel traitement ou médicament le prestataire prescrit-il ?
   2. Où le prestataire conseille-t-il à la famille de recevoir les testes/traitement/médicament ? (centre de santé, pharmacie privée, etc.)
   3. Qu'est-ce que le prestataire explique à la famille au sujet du traitement ?
   4. Comment la famille répond-elle à la recommandation de traitement ? Notez s'ils semblent satisfaits, confus, nerveux, inquiets ou résistants au traitement. Notez toutes les questions qu'ils posent.
4. Le prestataire recommande-t-il des visites de suivi ? Ou, demandent-ils à la famille de visiter une autre section de l'établissement avant de partir? (pour une prescription, un test, etc.)
   1. Décrivez la recommandation du prestataire. Quand et dans quelles circonstances le prestataire recommande-t-il le retour à l'établissement de santé ?
5. Le prestataire dit-il quoi que ce soit sur le fait de parler aux autres membres de la famille de la maladie, de l'alimentation ou de la nutrition ? Décrivez ce que le prestataire dit et comment la famille réagit.
6. Heure de clôture: _______________
